# The Toll-Like Receptor 5 Agonist Entolimod Mitigates Lethal Acute Radiation Syndrome in Non-Human Primates

**DOI:** 10.1371/journal.pone.0135388

**Published:** 2015-09-14

**Authors:** Vadim I. Krivokrysenko, Ilia A. Toshkov, Anatoli S. Gleiberman, Peter Krasnov, Inna Shyshynova, Ivan Bespalov, Ratan K. Maitra, Natalya V. Narizhneva, Vijay K. Singh, Mark H. Whitnall, Andrei A. Purmal, Alexander N. Shakhov, Andrei V. Gudkov, Elena Feinstein

**Affiliations:** 1 Cleveland BioLabs, Inc. (CBLI), Buffalo, New York, United States of America; 2 Armed Forces Radiobiology Research Institute (AFRRI), Bethesda, Maryland, United States of America; 3 Department of Cell Stress Biology, Roswell Park Cancer Institute (RPCI), Buffalo, New York, United States of America; Johns Hopkins Hospital, UNITED STATES

## Abstract

There are currently no approved medical radiation countermeasures (MRC) to reduce the lethality of high-dose total body ionizing irradiation expected in nuclear emergencies. An ideal MRC would be effective even when administered well after radiation exposure and would counteract the effects of irradiation on the hematopoietic system and gastrointestinal tract that contribute to its lethality. Entolimod is a Toll-like receptor 5 agonist with demonstrated radioprotective/mitigative activity in rodents and radioprotective activity in non-human primates. Here, we report data from several exploratory studies conducted in lethally irradiated non-human primates (rhesus macaques) treated with a single intramuscular injection of entolimod (in the absence of intensive individualized supportive care) administered in a mitigative regimen, 1–48 hours after irradiation. Following exposure to LD_50-70/40_ of radiation, injection of efficacious doses of entolimod administered as late as 25 hours thereafter reduced the risk of mortality 2-3-fold, providing a statistically significant (P<0.01) absolute survival advantage of 40–60% compared to vehicle treatment. Similar magnitude of survival improvement was also achieved with drug delivered 48 hours after irradiation. Improved survival was accompanied by predominantly significant (P<0.05) effects of entolimod administration on accelerated morphological recovery of hematopoietic and immune system organs, decreased severity and duration of thrombocytopenia, anemia and neutropenia, and increased clonogenic potential of the bone marrow compared to control irradiated animals. Entolimod treatment also led to reduced apoptosis and accelerated crypt regeneration in the gastrointestinal tract. Together, these data indicate that entolimod is a highly promising potential life-saving treatment for victims of radiation disasters.

## Introduction

Acute radiation syndrome (ARS) is the clinical manifestation of pathologies that develop soon after exposure to toxic doses of whole or partial-body ionizing radiation [1, 2]. ARS in humans has been well characterized [3] and described as several principal subsyndromes (depending on the absorbed radiation dose): hematopoietic (HP), gastrointestinal (GI) and cerebrovascular (CV). In humans, the HP and GI subsyndromes are induced by a broad range of total body irradiation (TBI) doses starting at 2 and 4 Gy, respectively, and hence, always co-exist at doses >4 Gy. Radiation doses in the 2–4 Gy range cause death primarily via severe HP injury, leading to increased risk of sepsis due to immunosuppression (stemming from granulo- and lymphopenia) and hemorrhagic events due to thrombocytopenia. With higher levels of radiation exposure (>4 Gy), GI tract damage has an increasing impact on general morbidity and accelerates the onset of death by further raising the risk of bleeding, dehydration and sepsis due to reduced integrity of the GI epithelium on the background of disabled immune and coagulation systems. While multi-organ failure associated with TBI doses leading to the CV subsyndrome of ARS (which develops at doses higher than 10–20 Gy) is practically incurable, the types and degrees of damage associated with isolated HP or combined HP and GI subsyndromes may be potentially treatable. However, there is currently no medical radiation countermeasure (MRC) approved specifically for prevention or treatment of any type of ARS.

MRCs can be generally classified into three major categories: radioprotectants (administered before exposure), radiomitigators (administered shortly after radiation exposure, prior to development of manifest ARS) and therapeutics (aimed for use during manifest ARS). A number of MRCs are currently being developed by various academic and industrial institutions (for recent comprehensive reviews, see [4–6]). Whereas the first two categories of MRCs are comprised of pharmaceuticals (small molecules or biologics), MRCs belonging to the third category are mostly represented by cell replacement therapies.

Radioprotectants are aimed at reduction of radiation damage and include anti-oxidants such as e.g., amifostine, vitamin E, and 5-Androstenediol (5-AED) [7–14] which reduce the concentration of toxic reactive oxygen species generated through radiation-induced ionization of organic and inorganic molecules in cells and tissues. Another principal opportunity to prevent radiation injury is to use inhibitors of apoptosis, the main cause of cell loss in radiosensitive organs [15]. Strong radioprotective properties of inhibitors of the proapoptotic p53 pathway [16, 17] and activators of antiapoptotic NF-κB signaling [18–20] are in line with this paradigm.

Radioprotectants can be useful under conditions of planned and predictable radiation exposure: for military personnel, first responders, and some categories of civilians. However, their value is relatively limited in realistic radiation disaster scenarios in which radiation exposure is unexpected and cannot be prepared for. Reduction of the scale of casualties in such situations requires the use of radiomitigative agents that are effective when administered after irradiation. This is not a trivial task since, in large part, the effects of radiation are rapid and irreversible. Therefore, for radiomitigation, the emphasis shifts away from antioxidants and inhibitors of apoptosis towards agents capable of promoting tissue regeneration and/or enabling the organism to survive temporary vulnerabilities associated with coagulopathy, immunosuppression, and loss of GI tract integrity.

In this regard, pharmacological activation of NF-κB, a key regulator of innate and adaptive immune responses [21, 22], seems to be an ideal approach for radiomitigation. In addition to suppressing apoptosis and inducing endogenous antioxidants (e.g., SOD2 and ferritin [23, 24]), activated NF-κB drives production of a large spectrum of secreted bioactive factors (e.g., cytokines, chemokines, and anti-infective molecules), many of which have ameliorative effects on pathologies caused by systemic genotoxic stresses and promote tissue regeneration [25]. In fact, a significant proportion of reported radiomitigators are capable of NF-κB activation. These include 5-AED [10, 11, 26], synthetic mimetics of mycoplasma lipopeptides that act via stimulation of Toll-like receptor (TLR) 2/6 heterodimers [19, 20]; IL-12 (HemaMax) [27–29] and a pharmacologically optimized recombinant derivative of the natural TLR5 agonist, bacterial flagellin (FliC), entolimod (previously known as CBLB502) [18], which is the main topic of this study.

Entolimod is being developed as MRC under the Animal Rule [30] specifically designed by the US Food and Drug Administration (FDA) as regulatory framework for testing experimental therapeutics whose assessment for efficacy is non-ethical in humans. Instead (in addition to safety trials in humans) such therapeutics are evaluated for efficacy in two animal species in which the model of the disease of interest as well as the response to the drug of interest closely model those in humans. Therefore, experiments in non-human primate (NHP) model of ARS that closely resembles human ARS [31] are considered obligatory. Here, we summarize the data on the radiomitigative efficacy of entolimod obtained in a series of exploratory studies performed in 164 NHPs. These experiments demonstrated that entolimod acts as a potent mitigator of radiation injury, capable of substantial (2–3 fold) and significant reduction of the risk of death when administered as single injection within 48 hours after lethal (LD_50-75/40_) TBI in the absence of additional intensive individualized supportive care. Reduced mortality from ARS caused by entolimod treatment was associated with reduced damage to and accelerated recovery of both HP and GI system tissues. These properties of entolimod make it a highly promising candidate MRC for use in realistic scenarios of radiation disasters, when victims cannot rely on a specialized clinical care for an extended period of time.

## Materials and Methods

### Entolimod drug substance and vehicle

Entolimod was expressed in *E*. *coli* and purified to >98% purity (at SynCo BioPartners, LLC, Amsterdam, The Netherlands) using a validated cGMP process involving 2-step (ion-exchange and hydrophobic interaction) chromatographic purification followed by endotoxin removal with a dedicated ion exchange column. Release testing indicates <100 EU/mg endotoxin, <5 ng/mg residual DNA, and <100 ng/mg host cell protein content in the entolimod drug product. Absence of additional contaminating TLR ligands was confirmed using specific TLR-expressing reporter cell lines (InvivoGen, San Diego, CA). The vehicle for entolimod was Dulbecco’s Phosphate-Buffered Saline (PBS; Gibco BRL, Life Technologies Inc., Grand Island, NY) in earlier studies and PBS-0.1% Tween 80 (O’Brien Pharmacy, Mission, KS) in later studies. Animals received a single injection of entolimod or vehicle in the quadriceps muscle, using a dose volume of 0.2 ml/kg, at 1, 4, 16, 25 or 48 hours after the end of irradiation.

### Animals

The care and use of nonhuman primates (*Macaca mulatta*, Chinese subspecies, from Sichuan Province) were in accordance with the principles outlined in the current Guide for the Care and Use of Laboratory Animals published by the National Institutes of Health, and the recommendations of the Weatherall report for The Use of Non-Human Primates in Research (December 2006).

All procedures for NHP studies were reviewed and approved by one or more of the following: the IACUC of Frontier Biosciences (Chengdu, Sichuan Province, China) (studies Rs-03, Rs-04, Rs-06, Rs-08, Rs-09, and Rs-14; IACUC protocol numbers B200609-P01, B200704-P01, B200718-P001, B200803-P002, B200903-P001, and B200901-P001); the IACUC of University of Illinois at Chicago (study Rs-22; IACUC protocol number 09–125); and the US Department of Defense Animal Care and Use Review Office (studies Rs-08, Rs-09, and Rs-22; ACURO protocol numbers CBMS-FY08-007, CBMS-FY08-008, and CBMS-FY09-022).

The NHP irradiation studies were conducted by Frontier Biosciences, Chengdu, Sichuan Province, China (studies Rs-03, Rs-04, Rs-06, Rs-08, Rs-09, and Rs-14) and UIC Toxicology Research Lab, Chicago, IL (Rs-22), Contract Research Organizations that are OLAW assured (assurance numbers A5723-01 and A3460-01, respectively) and AAALAC accredited. The NHP irradiation studies were approved under IACUC protocol numbers listed above. During the study, all efforts were made to minimize pain, distress, and suffering as stated in the approved IACUC protocols.

For studies conducted at Frontier Biosciences, rhesus macaques were obtained from Ping’an Animal Breeding and Research Base (Chengdu, Sichuan Province, China) or Suzhou Xishan Zhongke Laboratory Animal Co., Ltd (Suzhou, Jiangsu Province, China). For the study conducted at UIC TRL, rhesus macaques (Chinese subspecies) were obtained from Covance Laboratories (Madison, WI). At both CROs, 2–5 year old animals of both genders that weighed between 3 and 7 kg were used. The animals were research and irradiation naïve, clinically healthy, and certified to be free of specific pathogenic microorganisms (such as *Salmonella sp*., *Shigella sp*., *Mycobacterium tuberculosis*, cercopithecine herpesvirus type I (B virus), and *Toxoplasma gondii*). All animals received helminthicide treatment at their breeding facilities. Upon arrival at CROs, macaques were quarantined (typically for ≥3 weeks) while being subjected to daily cage-side observations, repeated laboratory testing (complete blood counts, serum biochemistry, blood coagulation, and electrocardiography) as well as body weight and temperature monitoring. During the pre-study period, the animals were also trained for being hand-caught and briefly restrained. Upon completion of the pre-study period, animals meeting pre-defined health criteria (consistently displaying normal clinical and laboratory parameters) were accepted into the study and randomly assigned to study groups based on their most recently measured body weight (even distribution per gender per weight). Approximately equal numbers of male and female animals were included in each study group. During the entire observation period (before and after irradiation), animals were housed in individual stainless steel cages in environment-controlled rooms with room temperature of 16–29°C, relative humidity of 30–70%, and a 12 hour light/dark cycle. In addition to fresh fruits and vegetables daily, animals were fed primate chow in studies at Frontier Biosciences (either prepared in-house or manufactured by Beijing Keaoxieli Feed Co. Ltd, China) or commercial certified primate biscuits in the study conducted at UIC TRL (Harlan Teklad Primate Diet 2055C). Fresh drinking water was provided *ad libitum*.

### Radiation sources and irradiation

In studies conducted at Frontier Biosciences, animals received 25 mg/kg pentobarbital sodium in the animal room to achieve mild anesthesia before transportation and an additional 8–10 mg/kg Ketamine injection during transit to the irradiation facility. For irradiation, sedated animals were restrained in plastic irradiation chairs. 5–11 animals were irradiated simultaneously, with balanced inclusion of animals from all study groups in each irradiation cohort. Male and female animals were irradiated separately. Animals were irradiated bilaterally using the cobalt-60 (^60^Co) gamma-ray sources located at Sichuan Atomic Energy Institute. The first source (used in studies Rs-03, Rs-04, Rs-06, and Rs-08) was a vertical bundle of vertically aligned ^60^Co rods. The second source (used in studies Rs-09 and Rs-14) was configured as a vertical rectangular array of vertically aligned ^60^Co rods. Dose rates in different experiments varied from ~0.8 to 1.1 Gy/min (0.94, 0.92, 0.83, 0.73, 1.06 and 1.03 Gy/min for studies Rs-03, Rs-04, Rs-06, Rs-08, Rs-09 and Rs-14, respectively) and animals received a total 6.5–6.75 Gy in-air dose (equivalent to 6.0–6.2 Gy midline dose for ~3–5 kg animals). Individual animal dosimetry was performed using thermoluminescent dosimeter (TLD) sets provided and evaluated by Global Dosimetry Solutions, Inc. Calibration of the radiation field of the source was performed periodically based on the Chinese National Standard [Standard Method for Using the Ferrous Sulfate (Fricke) Dosimeter to Measure Absorbed Dose in Water] GB139-89.

In the Study Rs-22 conducted at UIC TRL, animals were sedated with ketamine (10–20 mg/kg) and placed in plastic restraint boxes for the duration of transport and irradiation. Animals were irradiated using a rotating 6 MV LINAC source (Varian Clinac 2100EX) to a uniform total body in-air dose of 11 Gy, at a dose rate of 0.8±0.025 Gy/min (to achieve a 10.1 Gy midline dose). Dose measurements were made at the center of the cylindrical phantom (diameter = 8–10 cm) containing a PTW 31010 0.1cc Semiflex Ion chamber placed on the both sides of each animal. NanoDot dosimeters were used for measurement of individual surface doses.

### In-life procedures

All animals participating in studies with survival as the primary endpoint were observed for 40 days after TBI. During this period, cage-side observations were performed two or three times each day, at least 6 hours apart. Signs of morbidity and moribundity were recorded.

Blood was repeatedly collected from saphenous or cephalic veins for monitoring of complete blood counts (pre-dose, then almost every day on days 1–15, then every 3–4 days) and cytokine and entolimod levels (pre-dose, then at least at 1, 2, 4, 8, 24 hours post-dose). During blood collection, animals were briefly restrained without sedation. Body weights (at least once per week) and body temperature (at least twice per week) were also recorded. Food and fruit consumption was evaluated daily on a semiquantitative scale (good, fair, or poor). Following irradiation, no intensive individualized supportive care was provided other than oral rehydration, topical anti-infective treatment of lesions and general analgesia with fentanyl patches and/or buprenorphine when deemed necessary by the study veterinarian. No ketamine or opiates known to affect the immune system [32–34] were used in the study after entolimod treatment. For nutritional support, animals were provided with water soaked biscuits and extra amount of fruit.

### Terminal procedures

Moribund animals were subjected to euthanasia based on pre-specified criteria: severe weight loss (>20% loss of initial weight over a 3-day period); complete anorexia (for >3 days, with signs of deterioration); weakness and inability to obtain food or water (for >24 hours); complete unresponsiveness; low core body temperature (<35.9°C) following a period of febrile neutropenia; severe rapidly developing acute anemia (<40 g/L hemoglobin, < 13% hematocrit, and a drop of ≥7% in hematocrit between consecutive tests); and/or other signs of severe organ system dysfunction with a poor prognosis (as determined by a veterinarian). The euthanasia criteria were based on those generally recommended by veterinarian guidelines for studies involving terminal endpoints [35] with addition of 2 ARS-specific criteria recommended by Armed Forces Radiobiology Research Institute (AFRRI), relating to the rapid onset of acute anemia or drop in core body temperature following a period of febrile neutropenia. Similar criteria for moribund animal euthanasia were also applied by other groups conducting efficacy studies in the NHP model of ARS [27–29, 36, 37]. In studies with survival as the primary endpoint, all animals that survived to day 40 after TBI were subjected to scheduled euthanasia. In studies aimed at evaluation of gastrointestinal (GI) tract morphology, animals were euthanized at 8 hours or 5–7 days after TBI (depending on the study). All animals that were euthanized or found dead were subjected to gross pathology examination, with samples of bone marrow (sternum), spleen, thymus, lymph nodes, and GI tract segments collected for histological examination. In Study Rs-14, bone marrow aspirates were collected from iliac crests for colony forming assays.

### Histology

General histological analysis of organ samples was performed by light microscopy of paraffin sections (3–5 μm thick, 1–3 per organ or intestinal segment of each animal) stained with hematoxylin-eosin (H&E). Immunohistochemical detection of SOD2 expression, TUNEL staining and EdU incorporation analyses were performed in deparaffinized sections. The following antibodies were used for immunohistochemical evaluation: goat anti-SOD2 (N-20) pAb (sc-18503, Santa Cruz Biotechnology, Santa Cruz, CA); mouse anti-smooth muscle actin mAb conjugated with Cy3 (C6198, Sigma-Aldrich, St. Louis, MO); rabbit anti-phospho-histone H3 pAb (06–570, Millipore, Billerica, MA); and rabbit anti-neuro-specific tubulin beta III pAb (ab18207, Abcam, Cambridge, UK). The following reagents and kits were used histochemical evaluation: ApopTag Fluorescent In Situ Apoptosis Detection Kit (S7110, Chemicon, Millipore, Billerica, MA) for TUNEL detection, and azide-modified Alexa Fluor 488 (Invitrogen, Life Technologies, Grand Island, NY) for EdU incorporation. Samples were examined using a Zeiss AxioImager A1 microscope equipped an epifluorescent light source; images were captured with an AxioCam MRc digital camera and processed with a Zeiss Axio Imager Z1 microscope (Carl Zeiss, Germany).

The extent of morphologic alterations observed in histological sections was assessed by a blind semi-quantitative evaluation (see S1 Methods).

### Bone marrow colony forming assays

Analysis of total CFC numbers (which includes CFU-G, CFU-M, CFU-GM, CFU-GEMM, and BFU-E colonies) as well as separate analyses of BFU-E and CFU-Mk were performed using media and reagents from StemCell Technologies according to the manufacturer’s instructions (MethoCult, Cat. #28404; MegaCult-C Cat. #28413; StemCell Technologies, Vancouver, Canada). The number of colonies per 10^4^ live cells was calculated (after adjustment for hemodilution according to [38]).

### Laboratory and analytical assays

Complete blood counts (CBC) analysis was performed using automated blood cell counters (at Frontier Biosciences: Cell-Dyn 3700SL, Abbott, USA; at UIC TRL: Advia 120, Siemens Healthcare, USA). Cytokine levels in plasma (using K2EDTA as an anticoagulant) were determined using Luminex multiplex immunological assays at Armed Forces Radiobiology Research Institute (Bethesda, MD), Baylor Institute for Immunology Research (Dallas, TX), or Millipore Corporation (St. Charles, MO). In study Rs-03, levels of G-CSF, IL-4, IL-6, IL-10, and IFNγ were analyzed using human-targeted Fluorokine MAP assays from R&D Systems (Minneapolis, MN) and levels of IL-2, IL-3, IL-8, IL-12p70, and IP-10 were tested using Upstate (Temecula, CA) human-targeted Beadlyte Multi-Cytokine Flex assays. In studies Rs-09 and Rs-14, levels of G-CSF, IL-6, IL-8, and IFNγ were analyzed using Non-Human Primate Cytokine/Chemokine Milliplex Panel from Millipore, Inc. (Billerica, MA) and IL-10 was analyzed using human-targeted Fluorokine MAP Luminex assay from R&D Systems (Minneapolis, MN).

Entolimod levels in serum or plasma were determined at CBLI using a sandwich ELISA method employing proprietary entolimod-specific polyclonal antibodies.

### Data analysis

#### Statistical analysis

Numbers of surviving animals (at 40 days after irradiation) were compared pair-wise using Fisher’s exact test. Kinetics of mortality was compared between groups by Log rank test. For analysis of the effect of entolimod treatment on survival, the natural logarithm of *odds ratio of survival* (odds of survival in the treated group divided by that in the control group) was chosen as the metric. The odds associated with a probability p were defined as p / (1 –p). For a group of size n with 100% survival, odds were defined as (n − 0.5) / n; for groups with 0% survival odds were defined as 0.5 / n. Quantitative data were evaluated using Student’s t-test. All tests were two-sided. P-values <0.05 were considered statistically significant. Error bars in graphs represent standard errors (unless specified otherwise). GraphPad Prism 5.0 and Microsoft Excel 2007–2010 were used for most statistical analyses.

#### Calculation of days with Grade 4 cytopenia/anemia

Definition of a study day as cytopenic/anemic was based on actual values when available, or on imputed values for days when samples were not collected. Imputation was performed by linear interpolation over time between actual measurements, or by using the last observation carried forward between the day of last available measured value and the day of death. Percentage of live days with Grade 4 cytopenia/anemia was calculated as number of days with cytopenia/anemia divided by number of days the animal was alive during the 40-day observation period.

#### Area under the curve (AUC) values for cytokine and entolimod levels were calculated using the trapezoid rule

To eliminate the influence of differences in basal cytokine levels, AUC values were background-adjusted by subtracting the minimum observed factor concentration from all other concentrations before calculation.

## Results

### Treatment of NHPs with entolimod within 48 hours after lethal TBI significantly reduces the risk of death from ARS

To investigate the potency of entolimod in increasing survival of NHPs when administered after lethal TBI, a series of non-GLP studies in rhesus macaques were performed using a TBI dose range of LD_50/40—_LD_75/40_ (50–75% lethal over 40 days). This TBI dose range was chosen as being an approximate upper threshold at which exposed individuals would be at substantial risk of death, but might still be salvageable by medical therapy. This report presents the results generated within four survival experiments, designated Rs-03, Rs-06, Rs-09 and Rs-14, involving a total of 164 animals. The study groups for all 4 experiments are shown in Table 1. In all of these studies, the effects of intramuscular (i.m.) injection of entolimod were monitored for 40 days after irradiation. In addition to monitoring animal morbidity and mortality, multiple physiological parameters, blood cell counts, levels of elicited cytokines in peripheral blood (pharmacodynamics) and entolimod pharmacokinetics (PK) were evaluated. To prevent suffering, moribund animals were euthanized according to a predefined set of criteria (uniformly used in NHP studies described here, see Materials and Methods). No supportive care was provided other than oral rehydration (drinking water given *ad libitum*) and non-systemic treatment of external lesions. Study groups were composed of approximately equal numbers of male and female animals. Following irradiation, animals generally developed a clinical picture of ARS with typical prodromal and manifest illness features [31, 39].

**Table 1:** Efficacy of a single injection of entolimod in increasing 40-day survival of lethally irradiated NHPs when administered at different dose levels within 1–48 hours after TBI.

				40-day survival						Kinetics of mortality	
Study	Irradiation dose	Entolimod dose, μg/kg	Injection time(s) relative to TBI, h	Group size(n)	No. of survivors	% of survivors	Absolute survival increase %	P-value ^A^	Survival odds ratio vs. vehicle	Mean survival time ± SE, days	P-value ^B^
Rs-03	~LD_75/40_	0 (vehicle)	+1	10	2	20%	-	-	-	18.7±3.7	-
	(6.5 Gy)^C^	40	+1	10	7	70%	50%	0.07	9.33	30.8±4.8	0.06
Rs-06	~LD_75/40_	0 (vehicle)	+16	8	2	25%	-	-	-	23.3±3.8	-
	(6.5 Gy)^C^	40	+16	12	8	67%	42%	0.17	6.00	30.6±4.1	0.17
		40	+25	10	7	70%	45%	0.15	7.00	35.0±2.7	**0.02**
		40	+48	12	8	67%	42%	0.17	6.00	32.4±3.4	0.10
Rs-09	~LD_50/40_	0 (vehicle)	+1	18	9	50%	-	-	-	29.4±2.7	-
	(6.75 Gy)^D^	0.3	+1	18	12	67%	17%	0.50	2.00	32.0±2.7	0.44
		3	+1	18	14	78%	28%	0.16	3.50	35.7±2.0	0.07
		10	+1	18	17	94%	44%	**0.007**	17.00	39.2±0.8	**0.003**
Rs-14	~LD_50/40_	0 (vehicle)	+25	10	4	40%	-	-	-	24.5±4.3	-
	(6.75 Gy)^D^	10	+25	10	10	100%	60%	**0.01**	*28*.*50* ^G^	40.0±0.0	**0.004**
		40	+25	10	8	80%	40%	**0.17**	6.00	35.3±3.1	0.06
Pooled vehicle vs. ≥10 μg/kg entolimod, +25 h	~LD_50-75/40_	0 (vehicle)^E^	+1 - +25	46	17	37%	-	-	-	24.9±1.8	-
	(6.5–6.75 Gy)	≥10^F^	+25	30	25	83%	46%	**0.0001**	8.53	36.8±1.4	**0.0001**

^A^ P-value by Fisher's exact test (two-tailed) for comparisons vs. vehicle groups within individual studies or in pooled group analysis

^B^ P-value by Log rank test (two-tailed) for comparisons vs. vehicle groups within individual studies or in pooled group analysis

^C^ Source I: Sichuan Atomic Energy Institute, cylindrical bundle of Co-60 rods

^D^ Source II: Sichuan Atomic Energy Institute, vertical array of Co-60 rods

^E^ Vehicle-treated animals from studies Rs-03, Rs-06, Rs-09, and Rs-14

^F^ Entolimod-treated animals from studies Rs-06 and Rs-14

^G^ Survival odds and survival odds ratios adjusted due to 100% survival are shown in italics

*Note*: The frequency of moribund euthanasia was as follows: Study Rs-03–91% (1/11—found dead), Study Rs-06–100%; Study Rs-09–85% (3/20 –found dead); Rs-14–88% (1/8 –found dead). The likely cause of death in all the non-euthanized animals was acute hemorrhage.

**Fig 1:**
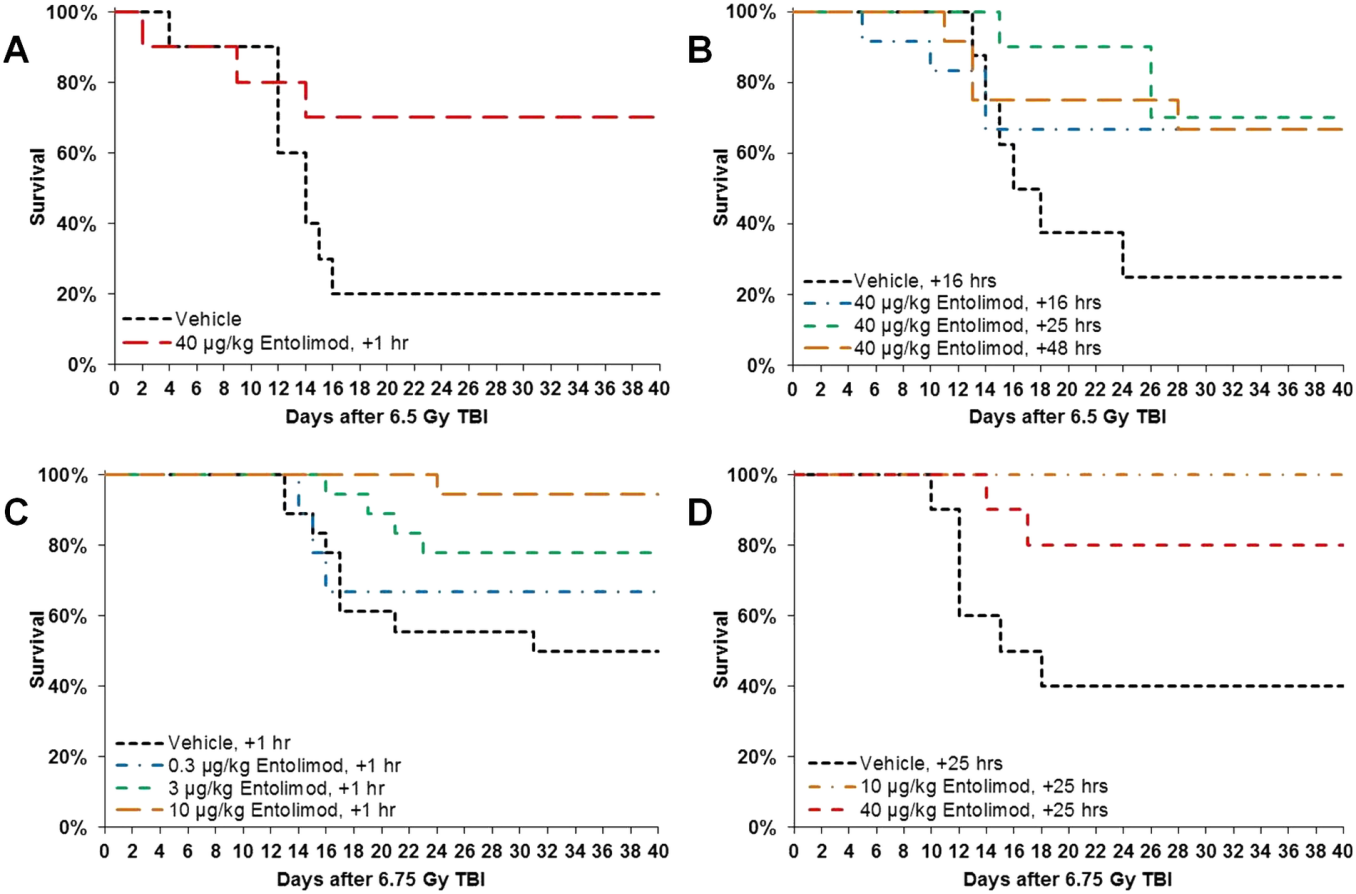
Improved survival of non-human primates (NHPs) injected with entolimod 1–48 hours after lethal irradiation. Kaplan-Meier plots of non-human primate (NHP) survival over the 40 days following exposure to LD_50/40_ –LD_75/40_ doses of total body irradiation (TBI) are shown. Time frame of entolimod efficacy (panels **A, B**) was evaluated in studies Rs-03 (treatment at 1 h after LD_75/40_ TBI; N = 10) and Rs-06 (treatment at 16, 25, or 48 h after LD_75/40_ TBI; N = 8–12). Dose-dependence of entolimod efficacy (panels **C, D**) was tested in studies Rs-09 (treatment at 1 h after LD_50/40_ TBI; N = 18) and Rs-14 (treatment at 25 h after LD_50/40_ TBI; N = 10).

First, in studies Rs-03 and Rs-06, we examined the effective timeframe for radiomitigative efficacy of 40 μg/kg entolimod, a dose previously established as radioprotective in NHPs [18]. Animals (n = 8–12 per group) were irradiated with 6.5 Gy TBI and treated with entolimod at 1, 16, 25, or 48 hours after irradiation. Forty-day survival in vehicle-treated groups was 20% and 25% in studies Rs-03 and Rs-06, respectively, whereas survival in all entolimod-treated groups was 67–75%. Thus, NHP survival was improved by an absolute 42–50% with survival odds ratios ranging from 6 to 9.3 regardless of entolimod administration time within the first 48 hours after TBI (Fig 1A and 1B; and Table 1).

The two subsequent studies, Rs-09 and Rs-14, explored dose-dependence of the survival effect of entolimod in NHPs at the boundaries of the 25-hour post-TBI period with the drug injected at either 1 or 25 hours after TBI, respectively. Animals (n = 10–18 per group) were irradiated with 6.75 Gy TBI (from a different radiation source compared to the two first studies) and received vehicle or entolimod injections at 0.3, 3 or 10 μg/kg (Rs-09) or 10 or 40 μg/kg (Rs-14) dose levels. Forty-day survival in vehicle-treated groups was 50% (Rs-09) or 40% (Rs-14), while entolimod doses of 10–40 μg/kg were fully efficacious in rescuing 80–100% of irradiated NHPs. These data correspond to increases in survival of 40–60% with survival odds ratios ranging between 6 and 28.5. Entolimod treatment at 3 μg/kg provided partial efficacy (28% survival increase) and the lowest tested dose of 0.3 μg/kg showed little or no efficacy (17% survival increase) (Fig 1C and 1D; and Table 1).

The survival advantage of 40–60% provided by efficacious entolimod doses was uniformly observed across all four of the studies reported here, although statistical significance was not reached in some individual groups (probably due to their small size). Similarity of study design elements, treatment regimens, and animal populations allowed meta-analysis in which similarly treated groups were pooled. Pooling of vehicle-treated groups from all 4 studies (n = 46) resulted in 37% 40-day survival, while pooling of all groups treated with fully efficacious 10 and 40 μg/kg entolimod doses at 25 hours after TBI (n = 30) indicated 83% 40-day survival. This 46% increase in 40-day survival was highly statistically significant (P = 0.0001 by Fisher’s exact test) with a survival odds ratio of 8.5 (Table 1).

The kinetics of mortality was similar in all 4 studies, with the majority of deaths occurring on days 12–16 after TBI and no deaths occurring after day 30. Mean survival time ranged from 18.7±3.7 to 29.4±2.7 days in vehicle-treated groups (24.9±1.8 days with all vehicle-treated groups pooled), while after ≥10 μg/kg entolimod treatment, mean survival time substantially increased to a range of 30.6±4.1 to 40.0±0.0 days (36.8±1.4 days in pooled group analysis; P = 0.0001 by Log-rank test for difference in survival kinetics) (Fig 1 and Table 1).

### Entolimod treatment accelerates recovery of hematopoiesis in lethally irradiated NHPs

Radiation damage to the HP system is one of the major causes of lethality at ~LD_50_-LD_70_ doses of TBI. Therefore, to investigate the mechanisms underlying entolimod’s radiomitigative efficacy, the content of different hematopoietic cell types was examined in peripheral blood and bone marrow samples collected in the four NHP studies described above.

**Fig 2:**
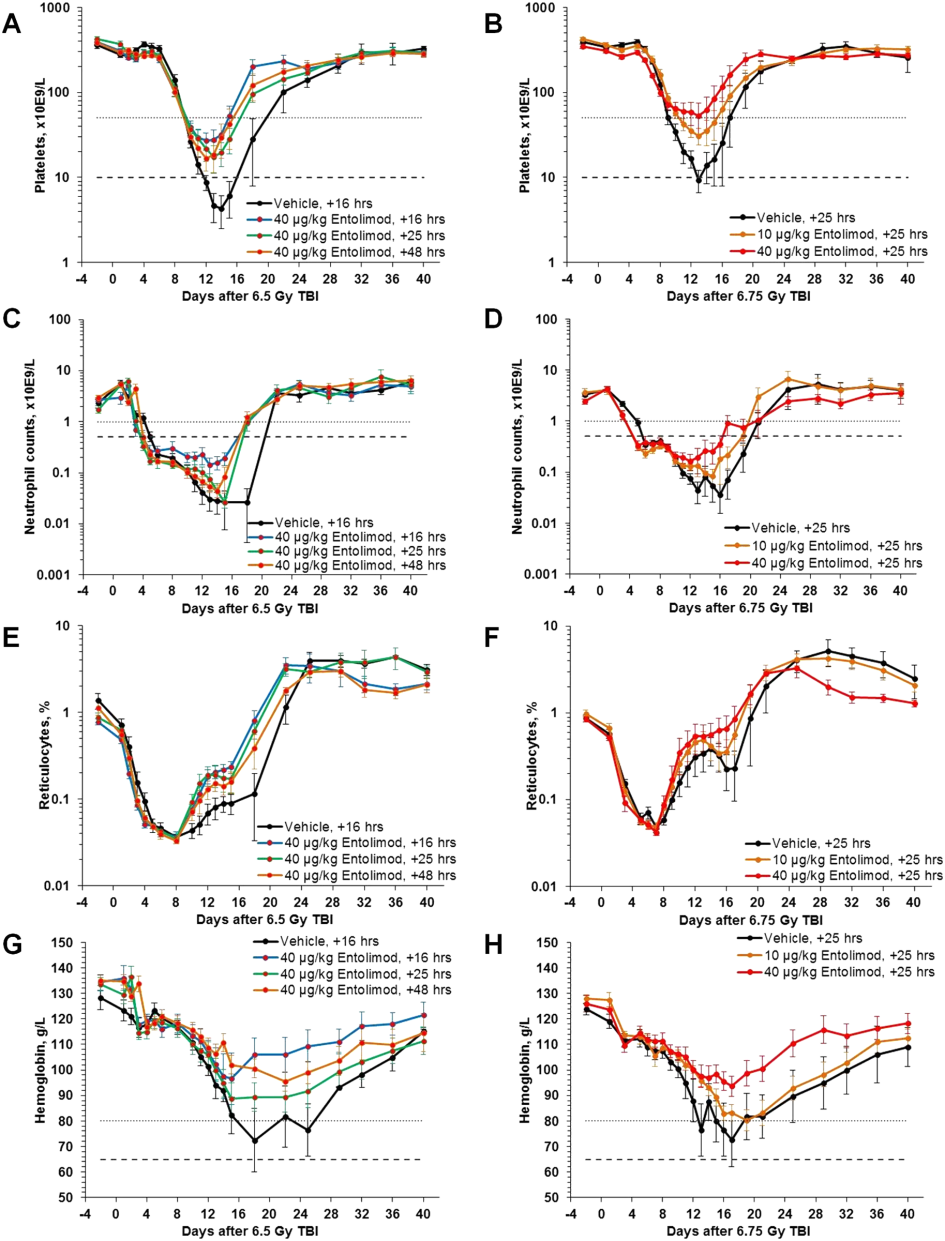
Accelerated hematological recovery of peripheral blood in NHPs injected with entolimod 16–48 hours post-irradiation. NHPs were treated with a single injection of entolimod at 16–48 hours after LD_50/40_ or LD_75/40_ TBI. **A, C, E, G**: Effect of 40 μg/kg entolimod administered at different time points (16, 25 or 48 hours) after 6.5 Gy TBI (LD_75/40_; study Rs-06; N = 8–12). **B, D, F, H**: Effect of different entolimod doses (10 or 40 μg/kg) administered at 25 hours after 6.75 Gy TBI (LD_75/40_; study Rs-14; N = 10). Cytopenia/anemia thresholds: dotted lines—Grade 3 (platelets <50,000/μL; neutrophils <1,000/μL; hemoglobin <80 g/L); dashed lines—Grade 4 (platelets <10,000/μL; neutrophils <500/μL; hemoglobin <65 g/L). Error bars represent standard errors.

Comparison of hematology data from control lethally irradiated NHPs and those treated with a single injection of entolimod revealed that across all four studies, the efficacious drug doses of ≥10 μg/kg reduced the duration and severity of thrombocytopenia, neutropenia, and anemia when given at any tested time point within 1–48 hours post-TBI (Fig 2A–2D and Fig 2G–2H, S1A–S1D Fig and S1G–S1H Fig; Table 2; and S1, S2, S3, and S4 Tables). In the context of ARS, anemia should be interpreted as a result of hemorrhage exacerbated by radiation-imposed suppression of compensating erythropoiesis (Fig 2E and 2F; S1E and S1F Fig) since mature erythrocytes are radioresistant [40] and their life span in the circulation of NHPs is 60 days [41].

**Table 2:** Mean nadir values of neutrophils, platelets and hemoglobin in peripheral blood following total body irradiation and vehicle or entolimod treatment.

					Neutrophils		Platelets		Hemoglobin	
Study	Irradiation dose	Entolimod dose, μg/kg	Injection time(s) relative to TBI, h	Group size (n)	Mean nadir ± SE (x10^3^/μL)	P-value ^A^	Mean nadir ± SE (x10^3^/μL)	P-value ^A^	Mean nadir ± SE (g/L)	P-value ^A^
Rs-03	~LD_75/40_	0 (vehicle)	+1	10	0.01±0.005	-	31±27.9	-	59.8±7.4	-
	(6.5 Gy)^B^	40	+1	10	0.18±0.128	0.20	59.6±26.2	0.46	77.8±6.9	0.09
Rs-06	~LD_75/40_	0 (vehicle)	+16	8	0.01±0.011	-	3.2±1.7	-	72.5±5.4	-
	(6.5 Gy)^B^	40	+16	12	0.06±0.017	**0.05**	34±16.4	0.09	92.9±7.4	**0.04**
		40	+25	10	0.02±0.007	0.51	15.8±5.9	0.07	83.3±5.4	0.18
		40	+48	12	0.02±0.005	0.85	12.5±4.6	0.08	93.1±3.2	**0.01**
Rs-09	~LD_50/40_	0 (vehicle)	+1	18	0.01±0.001	-	8±1.4	-	66.1±3.7	-
	(6.75 Gy)^C^	0.3	+1	18	0.01±0.003	0.44	7.2±1.2	0.66	69.1±3.5	0.56
		3	+1	18	0.02±0.006	**0.02**	16.6±3.7	**0.04**	78.9±4.1	**0.03**
		10	+1	18	0.03±0.009	**0.01**	22.4±3.9	**0.002**	76.7±3.7	**0.05**
Rs-14	~LD_50/40_	0 (vehicle)	+25	10	0.01±0.006	-	6.8±2.4	-	60.2±7.6	-
	(6.75 Gy)^C^	10	+25	10	0.04±0.011	0.11	21.8±5.4	**0.03**	77.5±3.8	0.06
		40	+25	10	0.07±0.029	0.09	39.9±12.4	**0.03**	89.6±4.4	**0.005**
Pooled vehicle vs. ≥10 μg/kg entolimod, +25 h	~LD_50-75/40_	0 (vehicle)^D^	+1–+25	46	0.01±0.003	-	11.9±6.1	-	64.6±2.9	-
	(6.5–6.75 Gy)	≥10^E^	+25	30	0.04±0.011	**0.006**	25.9±5.1	0.08	83.5±2.7	**<0.0001**

^A^ P-value by Student's t-test (two-tailed) for comparisons vs. vehicle groups within individual studies or in pooled group analysis

^B^ Source I: Sichuan Atomic Energy Institute, cylindrical bundle of Co60 rods

^C^ Source II: Sichuan Atomic Energy Institute, vertical array of Co60 rods

^D^ Vehicle-treated animals from studies Rs-03, Rs-06, Rs-09, and Rs-14

^E^ Entolimod-treated animals from studies Rs-06 and Rs-14

Although small group sizes reduced the statistical significance of the effects in some individual cases, the positive trends indicating entolimod-mediated amelioration of HP ARS were clearly observed in all reported studies (Table 2; and S1, S2, S3, and S4 Tables). In pooled group analysis (analogous to that described for survival), entolimod effects were highly statistically significant. Thus, nadir neutrophil counts were increased by entolimod treatment from 0.01±0.003 x10^3^/μl to 0.04±0.011 x10^3^/μl (P = 0.006), nadir platelet counts—from 11.9±6.1 x10^3^/μl to 25.9±5.1 x10^3^/μl (P = 0.08), and nadir hemoglobin levels—from 64.6±2.9 x10^3^/μl to 83.5±2.7 x10^3^/μl (P<0.0001) (Table 2). At the same time, the proportion of live days (when a particular animal was alive and had cytopenia) with Grade 4 neutropenia (neutrophil counts <500/μl) was decreased by entolimod treatment from 55%±3% in vehicle-treated groups to 41%±3% (P = 0.001, S1 Table), with Grade 4 thrombocytopenia (platelet counts <10,000/μl)–from 17%±2% to 5%±2% (P<0.0001, S3 Table), and with Grade 4 anemia (hemoglobin level <65 g/L)–from 10%±2% to 3%±2% (P = 0.003, S4 Table). While entolimod treatment did not change the incidence of Grade 4 neutropenia (proportion of animals that developed this condition at least once during 40 days of observation), it reduced the incidence of Grade 4 thrombocytopenia from 78% in vehicle-treated groups to 43% (pooled group analysis, P = 0.003, S3 Table), and of Grade 4 anemia—from 50% in control groups to 17% (pooled group analysis, P = 0.004, S4 Table). In addition to decreased severity and duration of thrombocytopenia, accelerated recovery of erythropoiesis in entolimod-treated NHPs (Fig 2E and 2F; S1E and S1F Fig) also contributed to markedly decreased incidence of ARS-associated Grade 4 anemia. Unlike the survival endpoint, where saturation of entolimod’s effect was achieved at dose of 10 μg/kg (Fig 1C and 1D), the effect of entolimod on hematological parameters continued to improve between 10 and 40 μg/kg doses (Fig 2B, 2D, 2F and 2H). This is consistent with the notion that achieving certain threshold levels (for example, above Grade 4 cytopenias/anemia) is sufficient to support survival.

**Fig 3:**
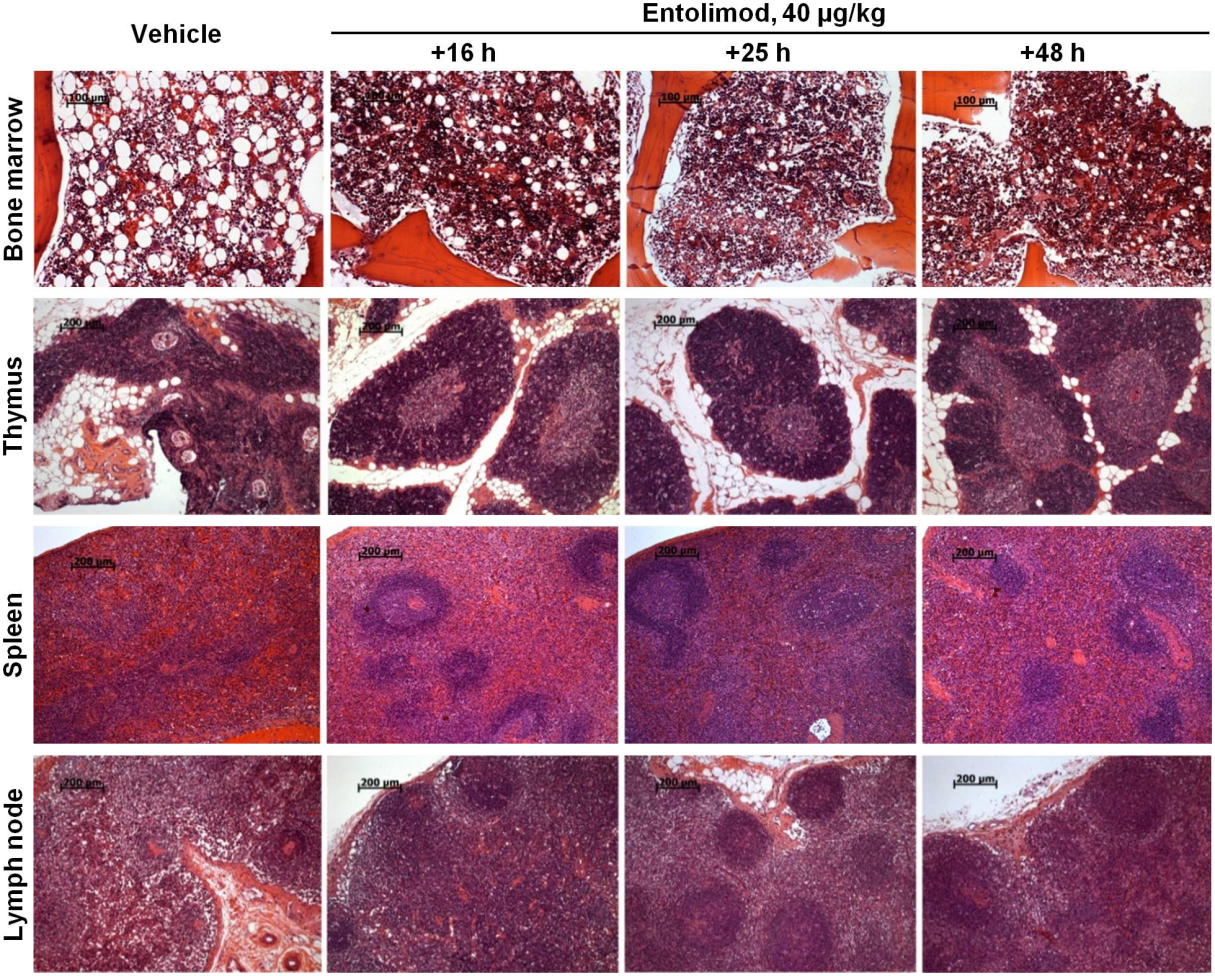
Enhanced morphological recovery of hematopoietic and lymphoid organs in NHPs treated with entolimod post-irradiation. NHPs were treated with a single injection of 40 μg/kg entolimod 16, 25 or 48 hours after LD_75/40_ total body irradiation (TBI). Tissue morphology was assessed 40 days post-irradiation and compared to that in control NHPs treated with vehicle 16 hours after LD_75/40_ TBI. Representative histological images (hematoxylin-eosin staining) of sternum bone marrow sections, thymuses, spleens and mesenteric lymph nodes of animals that survived to study termination on Day 40 post-TBI (study Rs-06) are shown. Scale bars: 100 μm for bone marrow, 200 μm for thymus, spleen, and lymph node.

Consistent with the finding of accelerated recovery of blood cellularity after hematopoietic nadirs in entolimod-treated irradiated NHPs, the bone marrow (BM) of treated animals displayed accelerated morphological recovery. Analysis of hematoxylin-eosin-stained sternum sections collected from surviving NHPs at 40 days post-TBI showed that BM from animals given a single injection of 40 μg/kg entolimod within 48 hours after TBI was considerably better regenerated compared to control monkeys. The hematopoietic cells were not only numerous, but also densely arranged in clusters among the sinusoids and the inconspicuous fat component. The elements of the three hematopoietic lineages (granulocytic, erythroid, and megakaryocytic) were spread out and in close contact with each other. In some animals, BM morphology was normal or close to normal, although others still had slightly or moderately hypoplastic BM. In contrast, BM of control NHPs was clearly less cellular and contained more adipose elements. Accelerated morphological recovery in entolimod-treated NHPs was also observed in lymphoid organs, including thymus, spleen and lymph nodes (Fig 3). These differences were statistically significant when blindly assigned semi-quantitative histological scores were compared (Table 3). Similar effects were induced by a single injection of 10 μg/kg entolimod administered at 25 hours after TBI (S2 Fig; and S5 Table).

**Table 3:** Histological evaluation of hematopoietic/lymphoid organs from NHPs that survived to day 40 after 6.5 Gy TBI and vehicle or entolimod treatment (study Rs-06)

Organ/tissue	Mean score ^A^ ± SE				P-value vs. vehicle ^B^		
	-	Entolimod, 40 μg/kg			Entolimod, 40 μg/kg		
	Vehicle (N = 2)	16 h (N = 8)	25 h (N = 7)	48 h (N = 8)	16 h	25 h	48 h
**Bone marrow**	1.3±0.3	3.4±0.1	3.6±0.2	3.4±0.2	**0.04**	**0.01**	**0.02**
**Thymus**	1.0±0.0	2.4±0.5	2.6±0.5	2.3±0.4	**0.02**	**0.02**	**0.02**
**Spleen**	1.0±0.0	2.0±0.3	1.7±0.4	1.5±0.2	**0.01**	0.14	**0.03**
**Lymph node**	1.0±0.0	1.6±0.3	1.9±0.3	2.0±0.2	**0.05**	**0.02**	**0.001**

^A^ Scoring was performed based on a 5-grade scale developed for each organ: 0 –total aplasia; 1 –pronounced atrophy, 2 –moderate atrophy, 3 –slight atrophy, close to normal morphology; 4 –normal morphology. Scoring criteria for individual organs are described in S1 Methods.

^B^ Student’s t-test vs. vehicle, 2-tailed.

To investigate the kinetics of entolimod-elicited BM recovery in more detail, a dedicated study was performed in which mice were injected with vehicle or entolimod 25 hours after 9 Gy TBI (~LD_50/30_) and then euthanized for histological and other evaluations at different time points. As shown in S3A Fig, the first histological signs of active hematopoiesis were evident in the BM of entolimod-treated mice as early as 3 days after TBI (2 days after drug treatment), with full-scale hematopoiesis observed by day 14. In comparison, the onset of hematopoietic recovery in vehicle-treated mice occurred between post-irradiation days 14 and 28, and full-scale hematopoiesis was further shifted to a time interval between days 28 and 56. Interestingly, the first signs of hematopoiesis in the BM of entolimod-treated mice were localized to the trabecular cell lining, suggesting stimulation of the HP stem cell compartment by entolimod [42]. Indeed, as early as 7 days after TBI, very early granulomonocytic progenitors (CFU-GM colonies) from entolimod-treated mice were elevated in number and displayed increased proliferative potential compared to those from vehicle-treated mice (S3B Fig).

Analysis of BM aspirates obtained from NHPs on day 40 after exposure to 6.75 Gy TBI and treated with either vehicle (n = 4) or 40 μg/kg entolimod (n = 4) 25 hours later revealed a clear positive influence of the drug on the content of hematopoietic progenitor cells, including total colony forming cells (CFC), erythroid burst forming units (BFU-E), and megakaryocyte colony forming units (CFU-Mk) (granulocyte lineage progenitors were not separately analyzed). The most substantial entolimod-elicited effect was on CFU-Mk, for which the frequency per 10^4^ viable BM cells was increased ~4.8-fold from 0.34 ±0.11 in vehicle-treated animals to 1.63 ±0.05 (P<0.05). The frequencies of CFC and BFU-E in entolimod-treated animals were increased by 22% and 36%, respectively, compared to vehicle-treated controls. The observed dominance of entolimod’s effect on CFU-Mk compared to progenitors of other lineages at this late time point (40 days after TBI) may be due to differences in the kinetics of recovery of different HP lineages, with the thrombopoietic lineage being known for its slow restoration following BM ablation and transplantation compared to other lineages [43].

Taken together, these data demonstrate that entolimod is a potent mitigator of radiation injury in the HP system, and acts via stimulation of accelerated hematopoietic recovery.

### Entolimod treatment reduces initial damage in the GI tract and accelerates its regeneration in lethally irradiated NHPs

Acute high-dose irradiation sufficient to induce GI ARS results in high degrees of apoptosis in the GI tract mucosa and submucosal elements (lamina propria, mucosa muscularis, lymphoid accumulations), leading to atrophy, increased permeability, susceptibility to hemorrhage (especially on the background of thrombocytopenia) and/or intussusceptions. Histological analyses were used to evaluate the effects of entolimod treatment on these signs of radiation damage to the NHP GI tract.

Assessment of NHP GI histology at 40 days after LD_50-75/40_ TBI doses did not reveal substantial differences between surviving entolimod- or vehicle-treated irradiated animals (S6 Table), most likely due to near-completion of regeneration by this late post-TBI time point regardless of treatment. Nevertheless, mean histological scores were generally higher in entolimod-treated groups compared to vehicle-treated control groups (S6 Table). This radiomitigative/pro-regeneration effect of entolimod was most apparent in the radiosensitive small intestine [44] and was observed in all histological substructures of the GI tract (villi and/or surface epithelium; crypts; and lamina propria with submucosa). The level of radiomitigation was moderate in the cecum and minimal to nonexistent in the colon and rectum, where radiation injury was not prominent (as observed in histological analysis of samples from animals that died during the course of the study).

To assess the effect of entolimod on the GI component of ARS during the peak of GI damage, three additional dedicated NHP studies were designed (S7 Table). In these studies, irradiated animals (a total of 48 NHPs, equal numbers of males and females) were euthanized at different time points between 8 hours and 7 days after TBI, when signs of immediate and early radiation-induced GI damage are typically observed along with indications of the initiation of recovery processes [18, 45–48]. The animals received TBI doses sufficient to induce moderate to severe GI injury (6.5–11 Gy, expected to cause 70–100% mortality) and entolimod doses ranging between 0.3 and 40 μg/kg at 1 to 25 hours after TBI. Some animals received EdU injections prior to euthanasia to allow evaluation of crypt proliferation by visualization of EdU incorporation on histological sections.

**Fig 4:**
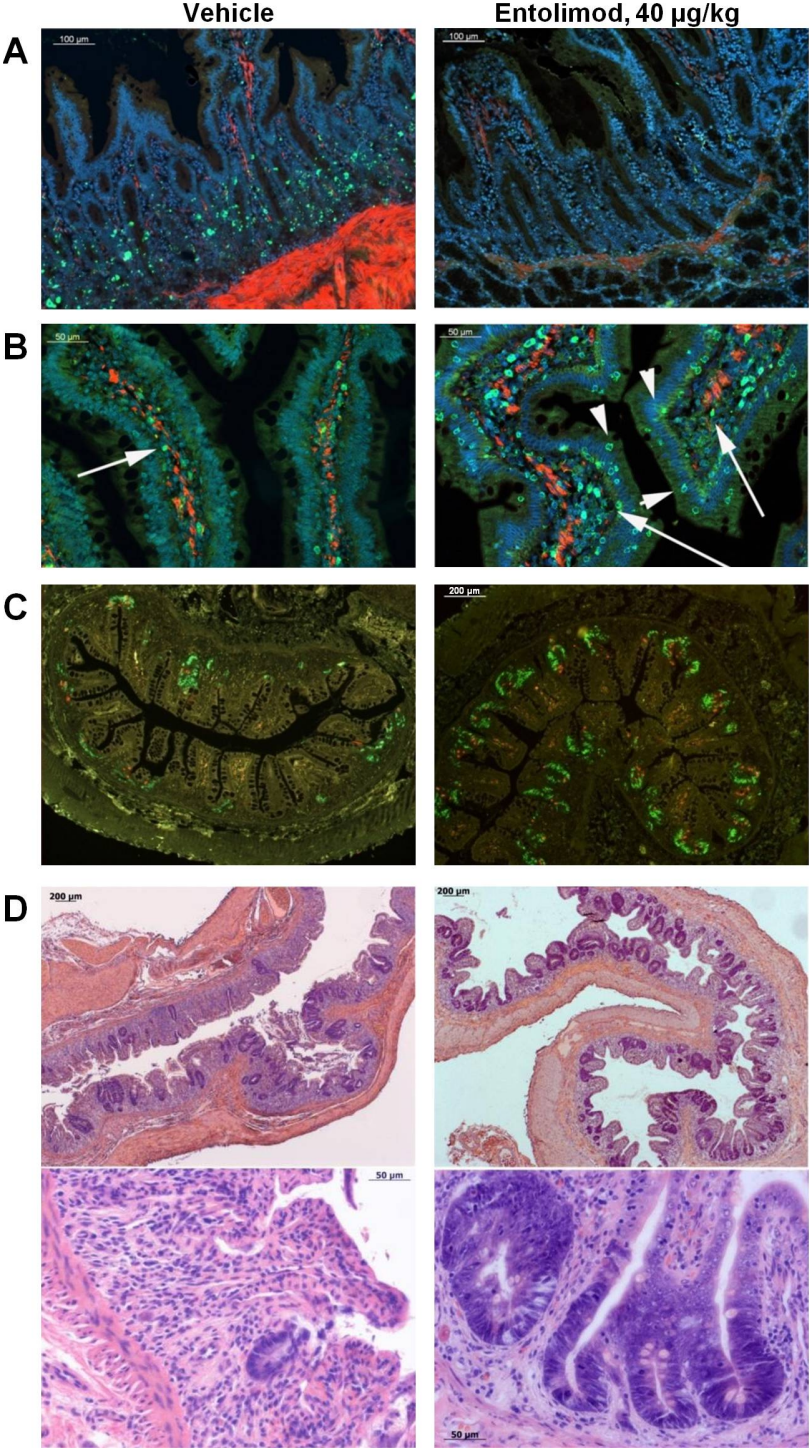
Entolimod treatment ameliorates radiation damage in the gastrointestinal (GI) tract. **A, B**. Small intestine sections from NHPs 8 hours after exposure to 6.5 Gy TBI and treatment with vehicle or 40 μg/kg entolimod 1 h later (study Rs-04). Blue—DAPI nuclear staining, red—smooth muscle actin immunostaining. **A**. TUNEL staining showing fewer apoptotic cells (green) in GI crypts of entolimod-treated NHPs (scale bar 100 μm); **B**. SOD2 immunostaining (green) showing more positive cells in GI villi (arrowheads) and lamina propria (arrows) of entolimod-treated NHPs (scale bar 50 μm). **C, D**. Small intestine sections of NHPs 7 days after exposure to 11 Gy TBI and treatment with vehicle or 40 μg/kg entolimod 4 h later (study Rs-22). **C**. Visualization of proliferating cells in the jejunum crypts: EdU (10 mg/kg i.v. 1 h before euthanasia) inclusion in replicating DNA (green) and phosphohistone 3 immunostaining of mitotic cells (red) showing more intensive proliferation of GI crypts in entolimod-treated NHPs (scale bar– 200 μm). **D**. H&E staining of ileum sections: upper panels—low magnification (scale bar– 200 μm), lower panels—high magnification (scale bar– 50 μm).

At 8 hours after 6.5 Gy TBI, the number of apoptotic cells counted in ~200 small intestine crypts was ~4.2-fold lower in animals treated with entolimod 1 hour after TBI (~1.16 TUNEL-positive cells/crypt) compared to vehicle-treated animals (~4.74 TUNEL-positive cells/crypt) (Fig 4A). There were only a few apoptotic cells in the crypts of the large intestine and rectum (~0.3 cells/crypt) regardless of treatment. Administration of entolimod also resulted in more robust expression of the NF-κB-regulated [23] anti-oxidant enzyme SOD2 in small intestine villi, crypts and lamina propria (Fig 4B). Entolimod-treated (1 hour after TBI) NHPs showed improved morphology in all analyzed GI segments at 5 days after exposure to 6.5 Gy TBI compared to vehicle-treated controls (S4 Fig). In addition to showing improved preservation/recovery of intestinal villi, crypts, and lymphoid accumulations in the lamina propria, entolimod-treated animals demonstrated better preservation of elements of the intestinal nervous system and muscularis mucosa (S5 Fig). The mitigative effect of entolimod on radiation-induced injury to the GI tract was inversely proportional to the time interval between irradiation and drug administration, but was still clearly observed even when the drug was given 25 hours after TBI (S4 Fig). At 7 days after 11 Gy TBI, irradiated and entolimod-treated (40 μg/kg, at 4 hours after TBI) NHPs, unlike vehicle-treated animals, demonstrated massive crypt regeneration as indicated by robust EdU incorporation (Fig 4C) and tissue morphology (microcolony growth visible by light microscopy, Fig 4D). Parallel semiquantitative blind histological assessment of radiation injury in different tissue elements throughout the small and large intestines showed statistically significant differences, indicating a beneficial effect of entolimod treatment (Table 4 and S8 Table).

**Table 4:** Histological evaluation of GI tract segments on day 7 after 11 Gy TBI and vehicle or 40 μg/kg entolimod treatment at +4 hours (study Rs-22, N = 4/group).

GI segment	Vehicle score ^A^, mean±SE	Entolimod score ^A^, mean±SE	T-test P-value (E vs. V) ^B^
**Oral Mucosa**	0.9±0.03	1.5±0.06	**0.001**
**Esophagus**	1.1±0.03	1.7±0.09	**0.01**
**Stomach**	1.1±0.05	2.3±0.08	**0.0001**
**Duodenum**	1.0±0.05	1.6±0.03	**0.0004**
**Jejunum**	0.9±0.07	1.3±0.12	0.05
**Ileum**	1.1±0.07	1.3±0.02	**0.04**
**Cecum**	1.1±0.07	1.3±0.03	0.08
**Ascending Colon**	0.9±0.17	1.5±0.15	**0.05**
**Transverse Colon**	1.0±0.17	1.4±0.02	0.08
**Descending Colon**	0.9±0.19	1.3±0.03	0.12
**Rectum**	1.1±0.03	1.2±0.05	0.15

^A^ 0: severely abnormal; 1: markedly abnormal; 2: moderately abnormal; 3: mildly abnormal; 4: normal (see S1 Methods).

^B^ Student’s t-test of entolimod (E) vs. vehicle (V) scores, 2-tailed

Overall, our data demonstrate that entolimod effectively mitigates radiation injury to the GI system, likely acting via reduction of apoptosis and stimulation of regeneration.

### Pharmacokinetics and pharmacodynamic effects of entolimod in lethally irradiated NHPs

**Fig 5:**
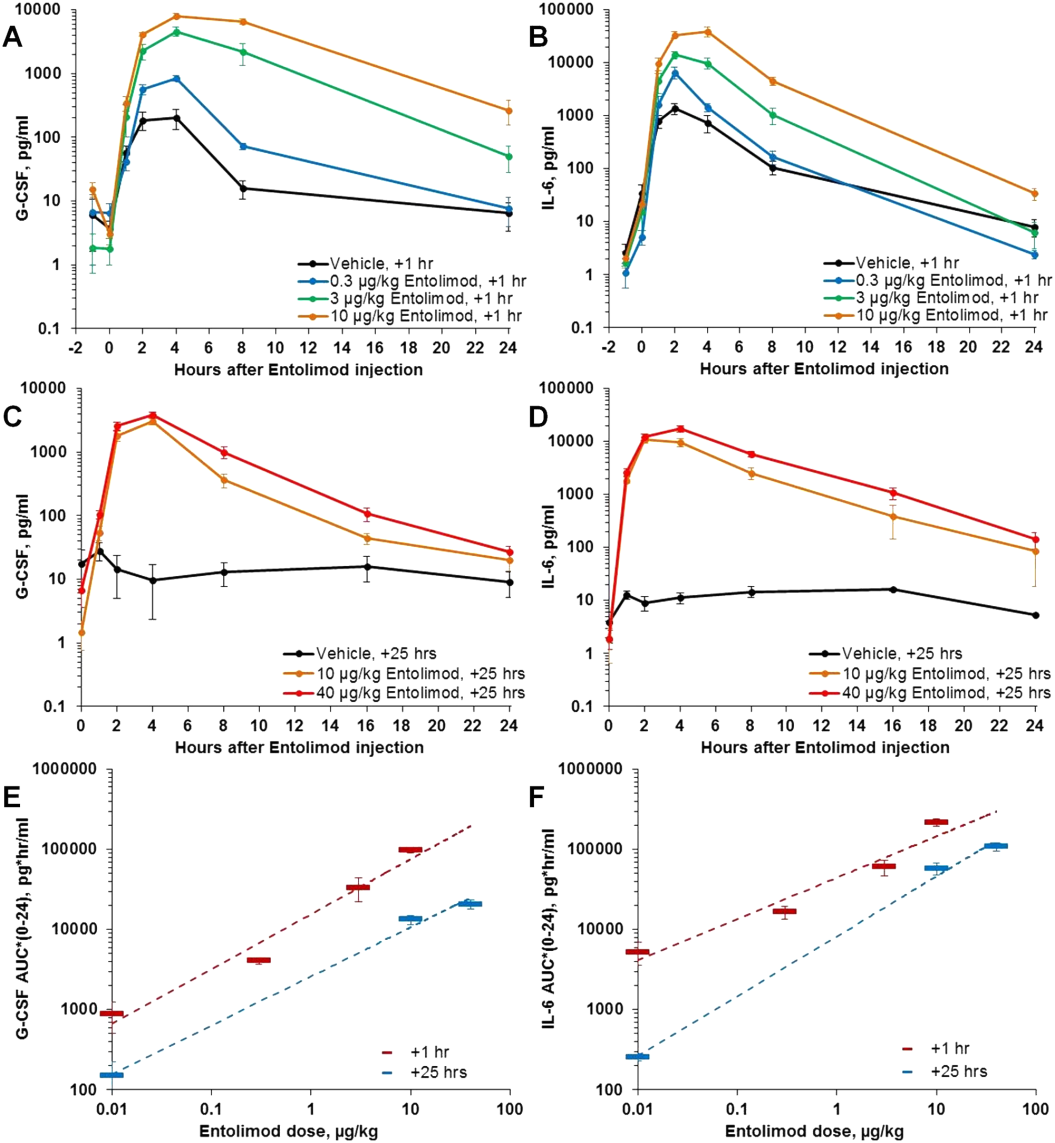
Effect of entolimod treatment on G-CSF and IL-6 levels in peripheral blood of irradiated NHPs. **A, B**: Effect of different entolimod doses administered 1 h after LD_50/40_ TBI (6.75 Gy; study Rs-09; N = 18). **C, D**: Effect of different entolimod doses administered 25 h after LD_50/40_ TBI (6.75 Gy; study Rs-14; N = 10). **E, F**: Comparison of dose-dependence of background-adjusted Area Under the Curve (AUC_0-24_) values for G-CSF and IL-6 after entolimod treatment given 1 h versus 25 h after LD_50/40_ TBI (with dashed log-linear regression lines). Error bars represent standard errors.

In the NHP studies reported here, plasma levels of numerous cytokines were measured at multiple time points following entolimod treatment. G-CSF and IL-6, previously established as potential entolimod efficacy biomarkers [49], displayed the most substantial and consistent dose-dependent responses to the drug when administered after LD_50-75/40_ TBI doses, with levels peaking on average at 2–4 hours after drug administration (Fig 5). These results are similar to observations made in non-irradiated NHPs and NHPs irradiated with LD_20-30/40_ TBI doses [49]. Both of these cytokines were induced somewhat by radiation alone (Fig 5A and 5B); therefore, treatment with entolimod close to TBI (e.g., at 1 hour after TBI) resulted in a combined effect of both treatments on the magnitude of cytokine increase. When entolimod was administered at 25 hours after TBI (after dissipation of radiation-induced G-CSF and IL-6 responses in vehicle-treated animals), entolimod-elicited cytokine profiles were more similar to those seen in non-irradiated animals (Fig 5C–5F and [49]).

Among other cytokines previously shown to be substantially influenced by entolimod in non-irradiated or sublethally irradiated (LD_20-30/40_ TBI) NHPs [49], IL-8, with neutrophil-mobilizing activity [50], and IL-10, with anti-inflammatory activity [51, 52], are also worth mentioning (S6 Fig). Both factors were found to be strongly responsive to entolimod and, to some extent, also to TBI. However, the magnitude of their elevation by entolimod and its dependence on drug dose were less consistent among different studies compared to G-CSF or IL-6. Neither radiation nor entolimod elicited any apparent response in pro-inflammatory cytokines such as IL-2, IP-10, IL-12p70, IL-4, IFNγ, or IL-3.

The pharmacokinetics of entolimod was similar in irradiated and non-irradiated NHPs, with C_max_ and AUC_0-24_ values being very close at identical drug doses. In both irradiated and non-irradiated NHPs, measured concentrations and exposures of entolimod in the blood displayed clear dose dependence (S7 Fig).

### Necropsy findings

Gross pathology findings at necropsy of animals that succumbed to ARS before Day 40 were generally consistent with those expected from ARS pathogenesis and consisted of hemorrhages (of varying extents and degrees of severity, mainly observed in the skin, GI tract, lungs and pericardium), septic complications (mainly in the lungs, pericardium, and skin), and generalized sepsis with multiple organ involvement. Among frequent findings, there were intussusceptions in the small and large intestines, adhesions in the abdominal and thoracic cavities, and signs of lung edema. There were no marked differences in gross pathology findings between entolimod- and vehicle-treated animals. This observation is not unexpected since entolimod treatment did not succeed in mitigating radiation damage in the animals that were necropsied during the course of the study, as evidenced by their mortality. At the same time, NHPs in which entolimod was actually effective (leading to their survival until study termination on Day 40 post-TBI—40–60% of animals at doses ≥ 10 μg/kg) could not be assessed for gross pathology status at the time of early recovery from ARS injury.

Gross pathology findings in animals that survived to the end of the study and were euthanized on Day 40–41 post-TBI were minimal regardless of treatment group, indicating that post-ARS recovery was generally complete by that time point. This observation was consistent with the lack of mortality from ARS after day 30 in all study groups. Certain differences indicating efficacy of entolimod, particularly in hematopoietic and lymphoid organs, were noticed at histological examination and are described in relevant sections above.

## Discussion

Development of an effective MRC is a cornerstone of the 2009 US Institute of Medicine report assessing medical preparedness to respond to a terrorist nuclear event and the 2009 US Homeland Security Council Planning Guidance to a Nuclear Detonation. A specific hypothetical scenario developed by Los Alamos Laboratories [53] for a 10KT ground explosion in Washington, DC estimates that ~130,000 individuals will receive between 1.25 and 8.3 Gy of irradiation. The chances of survival from lethal ARS for this group of victims could be increased if a relevant MRC were available. MRCs suitable for use in a mass-casualty scenario of this scale would ideally: (1) be effective even when administered late after TBI, as a single agent, and without the need for individualized intensive supportive care available only in hospitals; (2) be easily administered by untrained personnel; and (3) ameliorate both HP and GI damage (due to the interdependence of these two sub-syndromes).

The results described here and in previous publications [18, 49, 54–57] demonstrate that entolimod possesses all of the aforementioned desirable properties for an MRC. In fact, when given to NHPs as a single agent (without additional intensive supportive care) via a simple i.m. injection up to 48 hours after TBI, entolimod had a strong and consistent radiomitigative effect in four independent experiments involving 164 NHPs. Overall, entolimod treatment reduced the risk of NHP death 2-3-fold at TBI doses of LD_50-75/40_, providing an absolute survival advantage of 40–60% over vehicle treatment. In two additional studies with a total of 82 NHPs exposed to LD_20/40_-LD_30/40_ TBI doses, single injection of entolimod within at least 48 hours after TBI increased survival by 20–31%: from 69–80% in vehicle-treated control groups to 100% in entolimod-treated groups (V.I.K and E.F., unpublished data). Although the magnitude of achievable survival improvement in these latter two studies was limited by low lethality of the TBI doses used, the survival odds ratios were ≥4.75. The NHP studies reported here clearly show that entolimod treatment in the context of lethal TBI leads to reduced damage and accelerated recovery in both the radiosensitive HP and GI systems.

**Fig 6:**
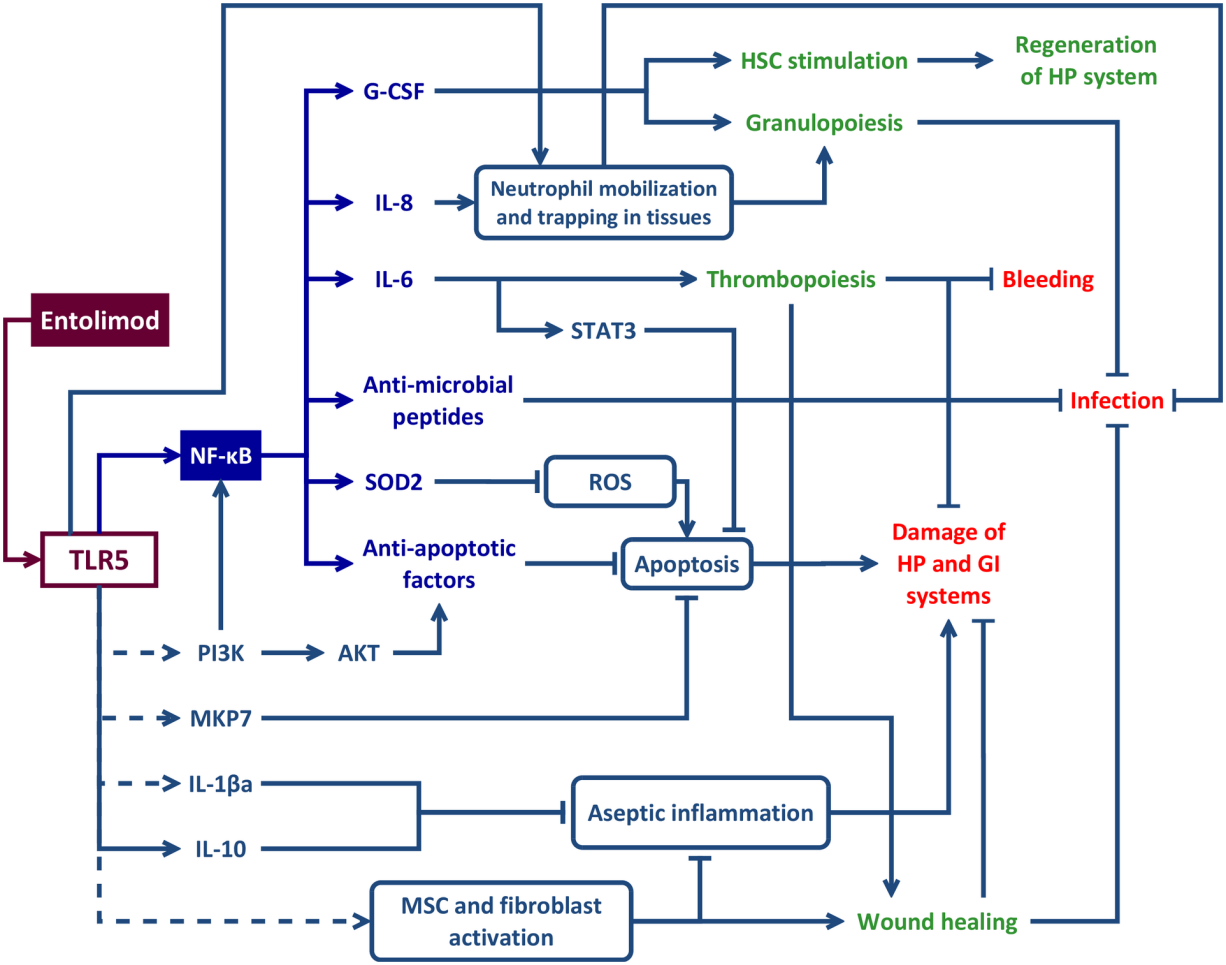
Schematic presentation of mechanism(s) underlying anti-acute radiation syndrome (ARS) effects of entolimod. Entolimod binding to Toll-like receptor 5 (TLR5) initiates a cascade of events, all merging at attenuation of major pathological processes—leading causes of death in ARS: damage to hematopoietic (HP) and gastrointestinal (GI) systems resulting in bleeding and sepsis. The immediate TLR5-dependent effectors include anti-oxidants (e.g., SOD2), anti-apoptotic factors (both NF-κB-dependent (i.e., IAP and Bcl family members [67–70]), and NF-κB-independent (i.e., PI3K/AKT, MKP7 and STAT3 [54, 71–73]), hematopoietic cytokines (e.g., G-CSF and IL-6 [49]), anti-infective factors [54, 90–95] and processes (e.g., neutrophil mobilization). In addition, stimulation of TLR5 is expected to inhibit radiation-induced aseptic inflammation involved in secondary tissue damage [64] e.g. via induction of an anti-inflammatory cytokine IL-10, IL-1β antagonist (IL-1βa) [76] and stimulation of mesenchymal stem cells (MSC) known to express TLR5 [77, 78] and to have anti-inflammatory properties [79]. Together with fibroblasts that can be induced to proliferate via TLR5 stimulation [104], MSC may also contribute to wound-healing processes [79]. Dashed lines show all molecular connections downstream of TLR5 that are not directly established for entolimod, but are extrapolated from published data on TLR5-dependent effects of flagellin.

Entolimod is a recombinant protein derived from the FliC flagellin of *Salmonella*, a natural ligand of TLR5 [58–60]. Expression of TLR5 on cells of both the HP and GI systems [61–63] was among the factors that initially prompted testing of its natural and engineered agonists as potential MRCs. Entolimod binds and stimulates TLR5 with the same specific activity as flagellin [18] and, hence, all known TLR5-mediated biological activities of flagellin can be theoretically also attributed to entolimod. The anti-ARS effects of both entolimod and FliC flagellin are entirely TLR5-dependent [49, 64]. Binding of entolimod to TLR5 results in stimulation of a number of downstream pathways, including those regulated by the key TLR5-activated transcription factor, NF-κB. Ultimately, as shown in the model in Fig 6, this engages multiple mechanisms of action against the multi-faceted toxic effects of ionizing radiation.

One of these mechanisms appears to be neutralization of radiation-triggered reactive oxygen species (ROS) by the enzyme superoxide dismutase (SOD2) [65], which is strongly induced by entolimod in both mouse [18] and NHP (this manuscript) models of ARS. Another mechanism that is a likely contributor to entolimod’s radioprotective and radiomitigative effects is inhibition of radiation-induced apoptosis, which is well-recognized as a major cause of the tissue damage and cytopenias observed in ARS [66]. Anti-apoptotic effects of entolimod are likely mediated via induction of NF-κB and its downstream anti-apoptotic effectors, such as members of the IAP [67] and Bcl-2 [68–70] protein families. Additional anti-apoptotic mechanisms of entolimod may include direct activation of the PI3K pathway [71, 72] and of a specific anti-apoptotic phosphatase, MKP7, recently identified as an inhibitor of radiation-dependent GI cell apoptosis [73], both triggered by flagellin stimulation of TLR5. In agreement with these published data, entolimod was shown to reduce radiation-induced apoptosis of cells in GI tissues both in mice [18] and NHPs (this manuscript). It may also attenuate apoptosis of other types of cells relevant to development of ARS. For example, inhibition of neutrophil apoptosis by flagellin treatment has been reported [74, 75]. In addition, stimulation of TLR5 is expected to inhibit radiation-induced aseptic inflammation involved in secondary apoptotic tissue damage [64] e.g. via induction of an anti-inflammatory cytokine IL-10, IL-1β antagonist (IL-1βa) [76] and stimulation of mesenchymal stem cells (MSC) known to express TLR5 [77, 78] and to have anti-inflammatory properties [79].

Restoration of the integrity and functionality of damaged organs following irradiation depends on the availability of sufficient numbers of undamaged tissue stem cells. The ability to protect and stimulate stem cells is expected to be an important property of any effective radiomitigator. In fact, our studies demonstrated protective and stimulatory effects of entolimod on stem cells in both HP and GI tissues as documented in irradiated or chemotherapy-treated mice [18, 57] and irradiated NHPs (this manuscript). Entolimod-treated irradiated NHPs displayed increased clonogenic potential of the BM and improved survival of intestinal-crypt stem cells as indicated by robust and accelerated crypt proliferation. The beneficial effects of entolimod on HP and GI stem cells are translated into facilitation of morphological recovery of the corresponding tissues [18, 56, 57]. The mechanism(s) underlying entolimod’s stimulatory effects on stem cells are likely mediated by induced cytokines, some of which are known to possess this activity [80–85]. Among the cytokines elevated in response to entolimod, two hematopoietic cytokines, G-CSF and IL-6, consistently showed the strongest induction. The importance of these cytokines for the radiomitigative activity of entolimod was proven experimentally *in vivo* using neutralizing antibodies [49] and is fully consistent with their previously defined biological activities as stimulators of granulo- and thrombopoiesis, respectively [86–88]. Consequently, both the severity and duration of radiation-induced thrombocytopenia and neutropenia were significantly reduced in entolimod-treated NHPs. Entolimod’s promotion of red blood cell lineage recovery (reticulocytes) with kinetics and magnitude similar to its effects on thrombocytopenia may be suggestive of stimulation of megakaryocyte/erythrocyte-restricted progenitors (MEPs), which in mouse experiments were shown to be sufficient to confer radioprotection [89]. The combined effects of entolimod on reducing the severity and duration of thrombocytopenia, and accelerating recovery of the erythroid lineage result in markedly diminished incidence of life-threatening Grade 4 hemorrhagic anemia, one of the hallmarks of HP ARS.

Loss of tissue integrity due to TBI leads to development of wounds and septic complications, which are especially dangerous on the background of impaired tissue repair and immunosuppression. Anti-infective properties reported for flagellin (and likely also relevant for entolimod due to its similar mechanism of action) are consistent with its general role as a trigger of TLR5-mediated innate immune response to bacterial infection. Indeed, flagellin was shown to induce secretion of antimicrobial factors, such as IL-17, S100A8/S100A9, hepcidin and other small peptides with antimicrobial activity, to support anti-infective defenses and tissue repair in the lungs, gut, skin and cornea [54, 90–95]. Direct anti-bacterial activity of flagellin (or a flagellin variant with a structure similar to that of entolimod) has been demonstrated in animal infection models [64, 93, 96]. This activity was likely associated with the ability of flagellin/entolimod to elicit early neutrophil mobilization (observed even in irradiated NHPs within 24 hours following entolimod treatment—see Fig 2C and 2D; S1C and S1D Fig) followed by neutrophil infiltration into tissues where they play an important role in local antibacterial responses [93]. Mobilization and tissue deposition of neutrophils (especially in the lung and the liver) can be explained by both entolimod-dependent induction of IL-8 [50] and by entolimod’s direct action on TLR5 expressed on the surface of neutrophils [97, 98]. TLR5 activation also enhances the phagocytic capacity and the respiratory burst activity of airway neutrophils, which likely contributes to their antibacterial potency [99]. At later times in the course of ARS, entolimod-induced accelerated recovery from radiation cytopenias would also be expected to contribute to antibacterial immunity (via restored granulocyte/macrophage function) and wound healing (via restored blood clotting and tissue trophic function of platelets) [100–103]. Another mechanism through which entolimod may promote wound healing is direct stimulation of fibroblasts and MSC [79, 104].

Many of the aforementioned factors and mechanisms involved in the anti-ARS activity of entolimod have been previously demonstrated to possess certain radiomitigative activities on their own. For example, AEOL 10150, a small molecule mimetic of antioxidant SOD, was shown to reduce local radiation-induced lung injury and increase survival of lethally-irradiated mice (although multiple administrations were required) [105, 106]. G-CSF-based Neupogen is in clinical use for treatment of chemotherapy-induced neutropenia. Moreover, G-CSF was recently shown to significantly improve survival of lethally irradiated NHPs if injected daily beginning 24 hours after TBI [36]. However, this effect was only achievable on the background of intensive supportive care [28] and was lost if drug administration was initiated 48 hours after TBI [37]. IL-6 is known to accelerate on its own and to potentiate G-CSF-mediated multi-lineage HP recovery following irradiation [107, 108]. Recombinant human IL-6 significantly improved recovery from chemotherapy-induced thrombocytopenia in clinical trials [109]. However, in a NHP model of sublethal (4.5 Gy) radiation-induced bone marrow aplasia, only a regimen of multiple daily IL-6 administrations starting from 24 hours after TBI and continuing for 23 consecutive days was able to boost platelet count restoration (with granulopoiesis being unaffected) [81]. These limitations make recombinant human IL-6 or Neupogen only marginally suitable for use in potential mass casualty radiation disasters. Hence, the power of entolimod as a radiomitigator, compared with products that address only a single aspect of radiation toxicity, is likely to stem from its pleiotropic mechanism of action allowing it to simultaneously counteract multiple aspects of radiation injury (summarized in Fig 6).

One of the candidate MRCs that is currently under development (by Neumedicines, Inc.) is IL-12/HemaMax. Similar to entolimod, a single injection of HemaMax administered subcutaneously 24 hours after lethal TBI was able to improve survival and hematopoietic recovery without additional supportive care in several experiments in NHPs [27–29]. However, the magnitude of its effects on cytopenias and survival was inferior to that of entolimod: absolute survival improvement reached with HemaMax at LD_50-65_ radiation doses was only 20% compared to 40–60% achieved with entolimod at LD_50-75_ doses of TBI, and did not reach statistical significance by pre-specified analyses [27, 28]. No data are available regarding HemaMax’s radioprotective activity when administered before TBI or radiomitigative activity when administered at time points later than 24 hours after TBI. IL-12 is known to act as a naïve T cell-activating factor promoting their Th1 maturation. It also stimulates production of cytotoxic cytokines such as IFNγ and TNFα from T-cells and natural killer (NK) cells, respectively [110]. Interestingly, neither IL-12 nor its principal downstream effector, IFNγ, was induced by entolimod in any species tested to date (humans, NHPs, rodents). Although the mechanism of action of HemaMax as a MRC has yet to be defined, it is clearly different from that of entolimod, which suggests that combination of the two compounds might result in additive or synergistic anti-ARS efficacy.

Entolimod is currently being developed towards approval as a MRC for reducing the risk of death following lethal TBI. Since it is not feasible or ethical to conduct trials in humans for evaluation of the efficacy of drugs against lethal TBI, formal development of entolimod is being conducted under the FDA’s Animal Efficacy Rule (21 C.F.R. $ 314.610) allowing pivotal demonstration of drug efficacy in accepted animal model(s) of a life-threatening human disease. The studies described in this manuscript resulted in determination of important parameters of entolimod activity (efficacious dose range and time window of administration relative to TBI) that allowed design and conduct of a pivotal efficacy experiment in the NHP model of ARS (submitted for publication) in support of FDA approval. In addition, these studies identified specific ameliorative effects of entolimod on HP and GI radiation injury, thus providing the mechanistic basis for its ability to significantly and substantially improve survival of lethally irradiated NHPs. An additional important finding of the reported experiments—that the anti-ARS effect of entolimod administered 48 hours after TBI is comparable to that at earlier time points—will be the focus of a future statistically powered time-ranging study to further define the extent of entolimod’s radiomitigative benefits.

## Supporting Information

S1 Fig Accelerated recovery of the peripheral blood cellularity and hemoglobin content in NHPs irradiated with LD_50/40_ or LD_75/40_ of TBI and treated with different doses of entolimod 1 hour later. (PDF)

S2 Fig Comparable restorative effects of single 10 or 40 μg/kg entolimod treatments given 25 hours after TBI on morphological recovery of hematopoietic and lymphoid organs in NHPs 40 days after irradiation with LD_50/40_ of TBI. (PDF)

S3 Fig Accelerated kinetics of bone marrow regeneration and proliferating phenotype of CFU-GM colonies in entolimod-treated CD2F1 mice after LD_50/30_ of TBI and entolimod treatment. (PDF)

S4 Fig Improved GI tract morphology in NHPs irradiated with 6.5 Gy TBI and treated with a single injection of entolimod at 1, 16, or 25 hours later. (PDF)

S5 Fig Improved preservation of intestinal innervation and muscularis mucosae integrity in the GI tract of irradiated NHPs treated with entolimod 1 hour after 6.5 Gy TBI. (PDF)

S6 Fig Effect of single dose entolimod treatment on IL-8 and IL-10 levels in the peripheral blood of NHPs irradiated with LD_50/40_ or LD_75/40_ doses of TBI. (PDF)

S7 Fig Entolimod concentrations in the peripheral blood of NHPs (irradiated with LD_50/40_ (6.75 Gy) TBI or non-irradiated) at different times after single intramuscular injection of the indicated drug doses. (PDF)

S1 Methods Semi-quantitative scoring of HP and GI organ morphology on histological sections. (PDF)

S1 Table Incidence and duration of Grade 4 neutropenia (neutrophil count <500 cells/μL) in lethally irradiated NHPs treated with vehicle or entolimod. (PDF)

S2 Table Incidence and duration of absolute neutropenia (neutrophil count <10 cells/μL) in lethally irradiated NHPs treated with vehicle or entolimod. (PDF)

S3 Table Incidence and duration of Grade 4 thrombocytopenia (platelet count <10,000 cells/μL) in lethally irradiated NHPs treated with vehicle or entolimod. (PDF)

S4 Table Incidence and duration of Grade 4 anemia (hemoglobin level <65g/L) in lethally irradiated NHPs treated with vehicle or entolimod. (PDF)

S5 Table Semi-quantitative histological evaluation of hematopoietic/lymphoid organs from NHPs that survived to day 40 after 6.75 Gy TBI followed by vehicle or entolimod treatment (study Rs-14). (PDF)

S6 Table Semi-quantitative histological evaluation of GI tract segments from NHPs that survived to day 40 after 6.5 Gy TBI and vehicle or entolimod treatment (study Rs-06). (PDF)

S7 Table Layout of studies dedicated to assessment of entolimod effects on GI tract histopathology in the course of ARS. (PDF)

S8 Table Semi-quantitative histological evaluation of GI tract sub-structures on day 7 after 11 Gy TBI and vehicle or 40 μg/kg entolimod treatment at 4 h post-TBI (study Rs-22, N = 4). (PDF)

## References

[1] DiCarlo AL, Maher C, Hick JL, Hanfling D, Dainiak N, Chao N, et al Radiation injury after a nuclear detonation: medical consequences and the need for scarce resources allocation. Disaster Med Public Health Prep. 2011;5 Suppl 1:S32–44. Epub 2011/03/22. 10.1001/dmp.2011.17 . 21402810 PMC3643117

[2] Waselenko JK, MacVittie TJ, Blakely WF, Pesik N, Wiley AL, Dickerson WE, et al Medical management of the acute radiation syndrome: recommendations of the Strategic National Stockpile Radiation Working Group. Ann Intern Med. 2004;140(12):1037–51. . 15197022 10.7326/0003-4819-140-12-200406150-00015

[3] Dorr H, Meineke V. Acute radiation syndrome caused by accidental radiation exposure—therapeutic principles. BMC Med. 2011;9:126 Epub 2011/11/26. 10.1186/1741-7015-9-126 . 22114866 PMC3235966

[4] Singh VK, Newman VL, Romaine PL, Wise SY, Seed TM. Radiation countermeasure agents: an update (2011–2014). Expert Opin Ther Pat. 2014;24(11):1229–55. Epub 2014/10/16. 10.1517/13543776.2014.964684 . 25315070 PMC4438421

[5] Singh VK, Romaine PL, Newman VL. Biologics as countermeasures for acute radiation syndrome: where are we now? Expert Opin Biol Ther. 2014:1–7. Epub 2014/11/25. 10.1517/14712598.2015.986453 . 25416452 PMC4720033

[6] Singh VK, Newman VL, Seed TM. Colony-stimulating factors for the treatment of the hematopoietic component of the acute radiation syndrome (H-ARS): a review. Cytokine. 2015;71(1):22–37. Epub 2014/09/13. 10.1016/j.cyto.2014.08.003 . 25215458

[7] Bensadoun RJ, Schubert MM, Lalla RV, Keefe D. Amifostine in the management of radiation-induced and chemo-induced mucositis. Support Care Cancer. 2006;14(6):566–72. . 16586122 10.1007/s00520-006-0047-4

[8] Singh PK, Wise SY, Ducey EJ, Brown DS, Singh VK. Radioprotective efficacy of tocopherol succinate is mediated through granulocyte-colony stimulating factor. Cytokine. 2011;56(2):411–21. Epub 2011/09/02. 10.1016/j.cyto.2011.08.016 . 21880504

[9] Srinivasan V, Weiss JF. Radioprotection by vitamin E: injectable vitamin E administered alone or with WR-3689 enhances survival of irradiated mice. Int J Radiat Oncol Biol Phys. 1992;23(4):841–5. Epub 1992/01/01. . 1319980 10.1016/0360-3016(92)90657-4

[10] Whitnall MH, Wilhelmsen CL, McKinney L, Miner V, Seed TM, Jackson WE 3rd. Radioprotective efficacy and acute toxicity of 5-androstenediol after subcutaneous or oral administration in mice. Immunopharmacol Immunotoxicol. 2002;24(4):595–626. . 12510793 10.1081/iph-120016038

[11] Stickney DR, Dowding C, Authier S, Garsd A, Onizuka-Handa N, Reading C, et al 5-androstenediol improves survival in clinically unsupported rhesus monkeys with radiation-induced myelosuppression. Int Immunopharmacol. 2007;7(4):500–5. . 17321473 10.1016/j.intimp.2006.12.005

[12] Gratwohl A, John L, Baldomero H, Roth J, Tichelli A, Nissen C, et al FLT-3 ligand provides hematopoietic protection from total body irradiation in rabbits. Blood. 1998;92(3):765–9. . 9680342

[13] Zsebo KM, Smith KA, Hartley CA, Greenblatt M, Cooke K, Rich W, et al Radioprotection of mice by recombinant rat stem cell factor. Proc Natl Acad Sci U S A. 1992;89(20):9464–8. . 1384054 10.1073/pnas.89.20.9464 PMC50152

[14] Dainiak N, Waselenko JK, Armitage JO, MacVittie TJ, Farese AM. The hematologist and radiation casualties. Hematology Am Soc Hematol Educ Program. 2003:473–96. . 14633795 10.1182/asheducation-2003.1.473

[15] Komarova EA, Gudkov AV. Chemoprotection from p53-dependent apoptosis: potential clinical applications of the p53 inhibitors. Biochem Pharmacol. 2001;62(6):657–67. Epub 2001/09/15. . 11556286 10.1016/s0006-2952(01)00733-x

[16] Komarov PG, Komarova EA, Kondratov RV, Christov-Tselkov K, Coon JS, Chernov MV, et al A chemical inhibitor of p53 that protects mice from the side effects of cancer therapy. Science. 1999;285(5434):1733–7. . 10481009 10.1126/science.285.5434.1733

[17] Strom E, Sathe S, Komarov PG, Chernova OB, Pavlovska I, Shyshynova I, et al Small-molecule inhibitor of p53 binding to mitochondria protects mice from gamma radiation. Nat Chem Biol. 2006;2(9):474–9. Epub 2006/07/25. 10.1038/nchembio809 . 16862141

[18] Burdelya LG, Krivokrysenko VI, Tallant TC, Strom E, Gleiberman AS, Gupta D, et al An agonist of toll-like receptor 5 has radioprotective activity in mouse and primate models. Science. 2008;320(5873):226–30. Epub 2008/04/12. 10.1126/science.1154986 . 18403709 PMC4322935

[19] Shakhov AN, Singh VK, Bone F, Cheney A, Kononov Y, Krasnov P, et al Prevention and mitigation of acute radiation syndrome in mice by synthetic lipopeptide agonists of Toll-like receptor 2 (TLR2). PLoS One. 2012;7(3):e33044 10.1371/journal.pone.0033044 22479357 PMC3314012

[20] Singh VK, Ducey EJ, Fatanmi OO, Singh PK, Brown DS, Purmal A, et al CBLB613: a TLR 2/6 agonist, natural lipopeptide of Mycoplasma arginini, as a novel radiation countermeasure. Radiat Res. 2012;177(5):628–42. Epub 2011/12/20. 10.1667/RR2657.1 . 22175300

[21] Dev A, Iyer S, Razani B, Cheng G. NF-kappaB and innate immunity. Curr Top Microbiol Immunol. 2011;349:115–43. Epub 2010/09/18. 10.1007/82_2010_102 . 20848362

[22] Hayden MS, West AP, Ghosh S. NF-kappaB and the immune response. Oncogene. 2006;25(51):6758–80. . 17072327 10.1038/sj.onc.1209943

[23] Xu Y, Kiningham KK, Devalaraja MN, Yeh CC, Majima H, Kasarskis EJ, et al An intronic NF-kappaB element is essential for induction of the human manganese superoxide dismutase gene by tumor necrosis factor-alpha and interleukin-1beta. DNA Cell Biol. 1999;18(9):709–22. . 10492402 10.1089/104454999314999

[24] Pham CG, Bubici C, Zazzeroni F, Papa S, Jones J, Alvarez K, et al Ferritin heavy chain upregulation by NF-kappaB inhibits TNFalpha-induced apoptosis by suppressing reactive oxygen species. Cell. 2004;119(4):529–42. . 15537542 10.1016/j.cell.2004.10.017

[25] Luo JL, Kamata H, Karin M. The anti-death machinery in IKK/NF-kappaB signaling. J Clin Immunol. 2005;25(6):541–50. Epub 2005/12/29. 10.1007/s10875-005-8217-6 . 16380818

[26] Xiao M, Inal CE, Parekh VI, Chang CM, Whitnall MH. 5-Androstenediol promotes survival of gamma-irradiated human hematopoietic progenitors through induction of nuclear factor-kappaB activation and granulocyte colony-stimulating factor expression. Mol Pharmacol. 2007;72(2):370–9. Epub 2007/05/03. 10.1124/mol.107.035394 . 17473057

[27] Basile LA, Ellefson D, Gluzman-Poltorak Z, Junes-Gill K, Mar V, Mendonca S, et al HemaMax, a recombinant human interleukin-12, is a potent mitigator of acute radiation injury in mice and non-human primates. PLoS One. 2012;7(2):e30434 Epub 2012/03/03. 10.1371/journal.pone.0030434 . 22383962 PMC3286478

[28] Gluzman-Poltorak Z, Vainstein V, Basile LA. Recombinant interleukin-12, but not granulocyte-colony stimulating factor, improves survival in lethally irradiated nonhuman primates in the absence of supportive care: Evidence for the development of a frontline radiation medical countermeasure. Am J Hematol. 2014 Epub 2014/05/24. 10.1002/ajh.23770 . 24852354

[29] Gluzman-Poltorak Z, Mendonca SR, Vainstein V, Kha H, Basile LA. Randomized comparison of single dose of recombinant human IL-12 versus placebo for restoration of hematopoiesis and improved survival in rhesus monkeys exposed to lethal radiation. J Hematol Oncol. 2014;7:31 Epub 2014/04/09. 10.1186/1756-8722-7-31 . 24708888 PMC4108131

[30] US Department of Health and Human Services. Approval of biological products when human efficacy studies are not ethical or feasible. 21 CFR 601.

[31] Dorr H, Lamkowski A, Graessle DH, Bennett A, Shapiro A, Farese AM, et al Linking the human response to unplanned radiation and treatment to the nonhuman primate response to controlled radiation and treatment. Health Phys. 2014;106(1):129–34. Epub 2013/11/28. . 24276556 10.1097/HP.0b013e3182a12de0 PMC3843145

[32] Welters ID, Hafer G, Menzebach A, Muhling J, Neuhauser C, Browning P, et al Ketamine inhibits transcription factors activator protein 1 and nuclear factor-kappaB, interleukin-8 production, as well as CD11b and CD16 expression: studies in human leukocytes and leukocytic cell lines. Anesth Analg. 2010;110(3):934–41. Epub 2010/02/27. . 20185670 10.1213/ANE.0b013e3181c95cfa

[33] Miller LS, Morita Y, Rangan U, Kondo S, Clemens MG, Bulkley GB. Suppression of cytokine-induced neutrophil accumulation in rat mesenteric venules in vivo by general anesthesia. Int J Microcirc Clin Exp. 1996;16(3):147–54. Epub 1996/05/01. . 8856389 10.1159/000179165

[34] Thomas J, Carver M, Haisch C, Thomas F, Welch J, Carchman R. Differential effects of intravenous anaesthetic agents on cell-mediated immunity in the Rhesus monkey. Clin Exp Immunol. 1982;47(2):457–66. Epub 1982/02/01. . 6978785 PMC1536550

[35] Sergio D. Animal Euthanasia In: Chow PKH, Ng RTH, Ogden BE, editors. Using Animal Models in Biomedical Research: A Primer for the Investigator Hackensack, NJ: World Scientific; 2008.

[36] Farese AM, Cohen MV, Katz BP, Smith CP, Gibbs A, Cohen DM, et al Filgrastim improves survival in lethally irradiated nonhuman primates. Radiat Res. 2013;179(1):89–100. Epub 2012/12/06. 10.1667/RR3049.1 . 23210705 PMC4562422

[37] Farese AM, Brown CR, Smith CP, Gibbs AM, Katz BP, Johnson CS, et al The Ability of Filgrastim to Mitigate Mortality Following LD50/60 Total-body Irradiation Is Administration Time-Dependent. Health Phys. 2014;106(1):39–47. Epub 2013/11/28. . 24276548 10.1097/HP.0b013e3182a4dd2c PMC3888641

[38] Holdrinet RS, von Egmond J, Wessels JM, Haanen C. A method for quantification of peripheral blood admixture in bone marrow aspirates. Exp Hematol. 1980;8(1):103–7. Epub 1980/01/01. . 7409031

[39] Williams JP, Brown SL, Georges GE, Hauer-Jensen M, Hill RP, Huser AK, et al Animal models for medical countermeasures to radiation exposure. Radiat Res. 2010;173(4):557–78. Epub 2010/03/26. 10.1667/RR1880.1 . 20334528 PMC3021126

[40] Maia GA, Reno Cde O, Medina JM, Silveira AB, Mignaco JA, Atella GC, et al The effect of gamma radiation on the lipid profile of irradiated red blood cells. Ann Hematol. 2014;93(5):753–60. Epub 2013/11/13. 10.1007/s00277-013-1944-5 . 24218190

[41] Rowe AW. Primates: models for red cell transfusion studies—cryopreservation and survival of transfused red cells in primates. J Med Primatol. 1994;23(8):415–25. Epub 1994/10/01. . 7602577 10.1111/j.1600-0684.1994.tb00130.x

[42] Dominici M, Rasini V, Bussolari R, Chen X, Hofmann TJ, Spano C, et al Restoration and reversible expansion of the osteoblastic hematopoietic stem cell niche after marrow radioablation. Blood. 2009;114(11):2333–43. Epub 2009/05/13. 10.1182/blood-2008-10-183459 . 19433859 PMC2745851

[43] Yamazaki R, Kuwana M, Mori T, Okazaki Y, Kawakami Y, Ikeda Y, et al Prolonged thrombocytopenia after allogeneic hematopoietic stem cell transplantation: associations with impaired platelet production and increased platelet turnover. Bone Marrow Transplant. 2006;38(5):377–84. Epub 2006/08/18. 10.1038/sj.bmt.1705444 . 16915226

[44] Potten CS, Merritt A, Hickman J, Hall P, Faranda A. Characterization of radiation-induced apoptosis in the small intestine and its biological implications. Int J Radiat Biol. 1994;65(1):71–8. Epub 1994/01/01. . 7905913 10.1080/09553009414550101

[45] Maj JG, Paris F, Haimovitz-Friedman A, Venkatraman E, Kolesnick R, Fuks Z. Microvascular function regulates intestinal crypt response to radiation. Cancer Res. 2003;63(15):4338–41. . 12907601

[46] Vigneulle RM, Rao S, Fasano A, MacVittie TJ. Structural and functional alterations of the gastrointestinal tract following radiation-induced injury in the rhesus monkey. Dig Dis Sci. 2002;47(7):1480–91. Epub 2002/07/27. . 12141804 10.1023/a:1015846514471

[47] Wilson JW, Pritchard DM, Hickman JA, Potten CS. Radiation-induced p53 and p21WAF-1/CIP1 expression in the murine intestinal epithelium: apoptosis and cell cycle arrest. Am J Pathol. 1998;153(3):899–909. Epub 1998/09/15. . 9736038 10.1016/S0002-9440(10)65631-3 PMC1853021

[48] Komarova EA, Kondratov RV, Wang K, Christov K, Golovkina TV, Goldblum JR, et al Dual effect of p53 on radiation sensitivity in vivo: p53 promotes hematopoietic injury, but protects from gastro-intestinal syndrome in mice. Oncogene. 2004;23(19):3265–71. . 15064735 10.1038/sj.onc.1207494

[49] Krivokrysenko VI, Shakhov AN, Singh VK, Bone F, Kononov Y, Shyshynova I, et al Identification of granulocyte colony-stimulating factor and interleukin-6 as candidate biomarkers of CBLB502 efficacy as a medical radiation countermeasure. J Pharmacol Exp Ther. 2012;343(2):497–508. Epub 2012/07/28. 10.1124/jpet.112.196071 . 22837010 PMC3477210

[50] van Eeden SF, Terashima T. Interleukin 8 (IL-8) and the release of leukocytes from the bone marrow. Leuk Lymphoma. 2000;37(3–4):259–71. . 10752978 10.3109/10428190009089427

[51] Hedrich CM, Bream JH. Cell type-specific regulation of IL-10 expression in inflammation and disease. Immunol Res. 2010;47(1–3):185–206. Epub 2010/01/21. 10.1007/s12026-009-8150-5 . 20087682 PMC2892196

[52] Ouyang W, Rutz S, Crellin NK, Valdez PA, Hymowitz SG. Regulation and functions of the IL-10 family of cytokines in inflammation and disease. Annu Rev Immunol. 2011;29:71–109. Epub 2010/12/21. 10.1146/annurev-immunol-031210-101312 . 21166540

[53] Benjamin G, McGeary MGH, McCutchen SR, Event. IoMUSCoMPfaTN. Assessing medical preparedness to respond to a terrorist nuclear event: workshop report. Washington, D.C.: National Academies Press; 2009 168 p. 25009935

[54] Burdelya LG, Brackett CM, Kojouharov B, Gitlin II, Leonova KI, Gleiberman AS, et al Central role of liver in anticancer and radioprotective activities of Toll-like receptor 5 agonist. Proc Natl Acad Sci U S A. 2013;110(20):E1857–66. Epub 2013/05/01. 10.1073/pnas.1222805110 . 23630282 PMC3657788

[55] Leigh ND, Bian G, Ding X, Liu H, Aygun-Sunar S, Burdelya LG, et al A flagellin-derived toll-like receptor 5 agonist stimulates cytotoxic lymphocyte-mediated tumor immunity. PLoS One. 2014;9(1):e85587 Epub 2014/01/24. 10.1371/journal.pone.0085587 . 24454895 PMC3891810

[56] Burdelya LG, Gleiberman AS, Toshkov I, Aygun-Sunar S, Bapardekar M, Manderscheid-Kern P, et al Toll-like receptor 5 agonist protects mice from dermatitis and oral mucositis caused by local radiation: implications for head-and-neck cancer radiotherapy. Int J Radiat Oncol Biol Phys. 2012;83(1):228–34. Epub 2011/10/18. 10.1016/j.ijrobp.2011.05.055 . 22000579 PMC3261342

[57] Kojouharov BM, Brackett CM, Veith JM, Johnson CP, Gitlin II, Toshkov IA, et al Toll-like receptor-5 agonist Entolimod broadens the therapeutic window of 5-fluorouracil by reducing its toxicity to normal tissues in mice. Oncotarget. 2014;5(3):802–14. Epub 2014/03/04. . 24583651 10.18632/oncotarget.1773 PMC3996654

[58] Anderson RF, Fisher LJ, Harris T. The 'pivotal antioxidant' hypothesis for the role of flavonoids in their reduction of HO* radical-induced damage on DNA. Redox Rep. 2001;6(3):197–9. Epub 2001/08/29. . 11523599 10.1179/135100001101536201

[59] Hayashi F, Smith KD, Ozinsky A, Hawn TR, Yi EC, Goodlett DR, et al The innate immune response to bacterial flagellin is mediated by Toll-like receptor 5. Nature. 2001;410(6832):1099–103. . 11323673 10.1038/35074106

[60] Smith KD, Andersen-Nissen E, Hayashi F, Strobe K, Bergman MA, Barrett SL, et al Toll-like receptor 5 recognizes a conserved site on flagellin required for protofilament formation and bacterial motility. Nat Immunol. 2003;4(12):1247–53. . 14625549 10.1038/ni1011

[61] Gewirtz AT, Navas TA, Lyons S, Godowski PJ, Madara JL. Cutting edge: bacterial flagellin activates basolaterally expressed TLR5 to induce epithelial proinflammatory gene expression. J Immunol. 2001;167(4):1882–5. . 11489966 10.4049/jimmunol.167.4.1882

[62] Feuillet V, Medjane S, Mondor I, Demaria O, Pagni PP, Galan JE, et al Involvement of Toll-like receptor 5 in the recognition of flagellated bacteria. Proc Natl Acad Sci U S A. 2006;103(33):12487–92. . 16891416 10.1073/pnas.0605200103 PMC1567905

[63] Means TK, Hayashi F, Smith KD, Aderem A, Luster AD. The Toll-like receptor 5 stimulus bacterial flagellin induces maturation and chemokine production in human dendritic cells. J Immunol. 2003;170(10):5165–75. Epub 2003/05/08. . 12734364 10.4049/jimmunol.170.10.5165

[64] Vijay-Kumar M, Aitken JD, Sanders CJ, Frias A, Sloane VM, Xu J, et al Flagellin treatment protects against chemicals, bacteria, viruses, and radiation. J Immunol. 2008;180(12):8280–5. Epub 2008/06/05. . 18523294 10.4049/jimmunol.180.12.8280

[65] Epperly MW, Sikora CA, DeFilippi SJ, Gretton JA, Zhan Q, Kufe DW, et al Manganese superoxide dismutase (SOD2) inhibits radiation-induced apoptosis by stabilization of the mitochondrial membrane. Radiat Res. 2002;157(5):568–77. Epub 2002/04/23. . 11966323 10.1667/0033-7587(2002)157[0568:msdsir]2.0.co;2

[66] Hendry JH, West CM. Apoptosis and mitotic cell death: their relative contributions to normal-tissue and tumour radiation response. Int J Radiat Biol. 1997;71(6):709–19. Epub 1997/06/01. . 9246185 10.1080/095530097143716

[67] Altieri DC. Survivin and IAP proteins in cell-death mechanisms. Biochem J. 2010;430(2):199–205. Epub 2010/08/14. 10.1042/BJ20100814 . 20704571 PMC3198835

[68] Sasi N, Hwang M, Jaboin J, Csiki I, Lu B. Regulated cell death pathways: new twists in modulation of BCL2 family function. Mol Cancer Ther. 2009;8(6):1421–9. Epub 2009/06/11. 10.1158/1535-7163.MCT-08-0895 . 19509269 PMC3091595

[69] Rolland SG, Conradt B. New role of the BCL2 family of proteins in the regulation of mitochondrial dynamics. Curr Opin Cell Biol. 2010;22(6):852–8. Epub 2010/08/24. 10.1016/j.ceb.2010.07.014 . 20729050 PMC2991415

[70] Wyllie AH. "Where, O death, is thy sting?" A brief review of apoptosis biology. Mol Neurobiol. 2010;42(1):4–9. Epub 2010/06/17. 10.1007/s12035-010-8125-5 . 20552413 PMC2894370

[71] Yu Y, Nagai S, Wu H, Neish AS, Koyasu S, Gewirtz AT. TLR5-mediated phosphoinositide 3-kinase activation negatively regulates flagellin-induced proinflammatory gene expression. J Immunol. 2006;176(10):6194–201. . 16670329 10.4049/jimmunol.176.10.6194

[72] Parcellier A, Tintignac LA, Zhuravleva E, Hemmings BA. PKB and the mitochondria: AKTing on apoptosis. Cell Signal. 2008;20(1):21–30. Epub 2007/08/25. 10.1016/j.cellsig.2007.07.010 . 17716864

[73] Jones RM, Sloane VM, Wu H, Luo L, Kumar A, Kumar MV, et al Flagellin administration protects gut mucosal tissue from irradiation-induced apoptosis via MKP-7 activity. Gut. 2011;60(5):648–57. Epub 2011/01/05. 10.1136/gut.2010.223891 . 21199832

[74] Francois S, El Benna J, Dang PM, Pedruzzi E, Gougerot-Pocidalo MA, Elbim C. Inhibition of neutrophil apoptosis by TLR agonists in whole blood: involvement of the phosphoinositide 3-kinase/Akt and NF-kappaB signaling pathways, leading to increased levels of Mcl-1, A1, and phosphorylated Bad. J Immunol. 2005;174(6):3633–42. Epub 2005/03/08. . 15749901 10.4049/jimmunol.174.6.3633

[75] Salamone GV, Petracca Y, Fuxman Bass JI, Rumbo M, Nahmod KA, Gabelloni ML, et al Flagellin delays spontaneous human neutrophil apoptosis. Lab Invest. 2010;90(7):1049–59. Epub 2010/04/07. 10.1038/labinvest.2010.77 . 20368700

[76] Carvalho FA, Aitken JD, Gewirtz AT, Vijay-Kumar M. TLR5 activation induces secretory interleukin-1 receptor antagonist (sIL-1Ra) and reduces inflammasome-associated tissue damage. Mucosal Immunol. 2011;4(1):102–11. Epub 2010/09/17. 10.1038/mi.2010.57 . 20844479 PMC3012739

[77] He XX, Bai H, Yang GR, Xue YJ, Su YN. [Expression of Toll-like receptors in human bone marrow mesenchemal stem cells]. Zhongguo Shi Yan Xue Ye Xue Za Zhi. 2009;17(3):695–9. Epub 2009/06/25. . 19549390

[78] van den Berk LC, Jansen BJ, Siebers-Vermeulen KG, Netea MG, Latuhihin T, Bergevoet S, et al Toll-like receptor triggering in cord blood mesenchymal stem cells. J Cell Mol Med. 2009. Epub 2009/02/03. 10.1111/j.1582-4934.2009.00653.x PMC4516497 20196781

[79] Garcia-Castro J, Trigueros C, Madrenas J, Perez-Simon JA, Rodriguez R, Menendez P. Mesenchymal stem cells and their use as cell replacement therapy and disease modelling tool. J Cell Mol Med. 2008;12(6B):2552–65. Epub 2009/02/13. 10.1111/j.1582-4934.2008.00516.x . 19210755 PMC3828873

[80] Neta R, Perlstein R, Vogel SN, Ledney GD, Abrams J. Role of interleukin 6 (IL-6) in protection from lethal irradiation and in endocrine responses to IL-1 and tumor necrosis factor. J Exp Med. 1992;175(3):689–94. Epub 1992/03/01. . 1311016 10.1084/jem.175.3.689 PMC2119144

[81] MacVittie TJ, Farese AM, Patchen ML, Myers LA. Therapeutic efficacy of recombinant interleukin-6 (IL-6) alone and combined with recombinant human IL-3 in a nonhuman primate model of high-dose, sublethal radiation-induced marrow aplasia. Blood. 1994;84(8):2515–22. Epub 1994/10/15. . 7919369

[82] Farrell CL, Bready JV, Rex KL, Chen JN, DiPalma CR, Whitcomb KL, et al Keratinocyte growth factor protects mice from chemotherapy and radiation-induced gastrointestinal injury and mortality. Cancer Res. 1998;58(5):933–9. . 9500453

[83] MacVittie TJ, Farese AM, Smith WG, Baum CM, Burton E, McKearn JP. Myelopoietin, an engineered chimeric IL-3 and G-CSF receptor agonist, stimulates multilineage hematopoietic recovery in a nonhuman primate model of radiation-induced myelosuppression. Blood. 2000;95(3):837–45. Epub 2000/01/29. . 10648394

[84] Bertho JM, Frick J, Prat M, Demarquay C, Dudoignon N, Trompier F, et al Comparison of autologous cell therapy and granulocyte-colony stimulating factor (G-CSF) injection vs. G-CSF injection alone for the treatment of acute radiation syndrome in a non-human primate model. Int J Radiat Oncol Biol Phys. 2005;63(3):911–20. Epub 2005/05/26. 10.1016/j.ijrobp.2005.03.045 . 15913916

[85] Dainiak N. Rationale and recommendations for treatment of radiation injury with cytokines. Health Phys. 2010;98(6):838–42. Epub 2010/05/07. . 20445391 10.1097/HP.0b013e3181b3fce5

[86] Clark SC, Kamen R. The human hematopoietic colony-stimulating factors. Science. 1987;236(4806):1229–37. Epub 1987/06/05. . 3296190 10.1126/science.3296190

[87] Roberts AW. G-CSF: a key regulator of neutrophil production, but that's not all!. Growth Factors. 2005;23(1):33–41. . 16019425 10.1080/08977190500055836

[88] Zeidler C, Kanz L, Hurkuck F, Rittmann KL, Wildfang I, Kadoya T, et al In vivo effects of interleukin-6 on thrombopoiesis in healthy and irradiated primates. Blood. 1992;80(11):2740–5. . 1280477

[89] Na Nakorn T, Traver D, Weissman IL, Akashi K. Myeloerythroid-restricted progenitors are sufficient to confer radioprotection and provide the majority of day 8 CFU-S. J Clin Invest. 2002;109(12):1579–85. Epub 2002/06/19. 10.1172/JCI15272 . 12070305 PMC151014

[90] Abtin A, Eckhart L, Glaser R, Gmeiner R, Mildner M, Tschachler E. The antimicrobial heterodimer S100A8/S100A9 (calprotectin) is upregulated by bacterial flagellin in human epidermal keratinocytes. J Invest Dermatol. 2010;130(10):2423–30. Epub 2010/06/18. 10.1038/jid.2010.158 . 20555353

[91] Gao N, Kumar A, Jyot J, Yu FS. Flagellin-induced corneal antimicrobial peptide production and wound repair involve a novel NF-kappaB-independent and EGFR-dependent pathway. PLoS One. 2010;5(2):e9351 Epub 2010/03/03. 10.1371/journal.pone.0009351 . 20195469 PMC2829077

[92] Kumar A, Gao N, Standiford TJ, Gallo RL, Yu FS. Topical flagellin protects the injured corneas from Pseudomonas aeruginosa infection. Microbes Infect. 2010;12(12–13):978–89. Epub 2010/07/06. 10.1016/j.micinf.2010.06.007 . 20601077 PMC3191947

[93] Munoz N, Van Maele L, Marques JM, Rial A, Sirard JC, Chabalgoity JA. Mucosal administration of flagellin protects mice from Streptococcus pneumoniae lung infection. Infect Immun. 2010;78(10):4226–33. Epub 2010/07/21. 10.1128/IAI.00224-10 . 20643849 PMC2950348

[94] Van Maele L, Carnoy C, Cayet D, Songhet P, Dumoutier L, Ferrero I, et al TLR5 signaling stimulates the innate production of IL-17 and IL-22 by CD3(neg)CD127+ immune cells in spleen and mucosa. J Immunol. 2010;185(2):1177–85. Epub 2010/06/23. 10.4049/jimmunol.1000115 . 20566828 PMC3060348

[95] Yu FS, Cornicelli MD, Kovach MA, Newstead MW, Zeng X, Kumar A, et al Flagellin stimulates protective lung mucosal immunity: role of cathelicidin-related antimicrobial peptide. J Immunol. 2010;185(2):1142–9. Epub 2010/06/23. 10.4049/jimmunol.1000509 . 20566829 PMC3038689

[96] Zgair AK. Flagellin administration protects respiratory tract from Burkholderia cepacia infection. J Microbiol Biotechnol. 2012;22(7):907–16. Epub 2012/05/15. . 22580309 10.4014/jmb.1112.11079

[97] Silva SC, Baggio-Zappia GL, Brunialti MK, Assuncao MS, Azevedo LC, Machado FR, et al Evaluation of Toll-like, chemokine, and integrin receptors on monocytes and neutrophils from peripheral blood of septic patients and their correlation with clinical outcomes. Braz J Med Biol Res. 2014;47(5):384–93. Epub 2014/04/15. . 24728213 10.1590/1414-431X20143190 PMC4075306

[98] Janot L, Sirard JC, Secher T, Noulin N, Fick L, Akira S, et al Radioresistant cells expressing TLR5 control the respiratory epithelium's innate immune responses to flagellin. Eur J Immunol. 2009;39(6):1587–96. Epub 2009/05/09. 10.1002/eji.200838907 . 19424969

[99] Koller B, Kappler M, Latzin P, Gaggar A, Schreiner M, Takyar S, et al TLR expression on neutrophils at the pulmonary site of infection: TLR1/TLR2-mediated up-regulation of TLR5 expression in cystic fibrosis lung disease. J Immunol. 2008;181(4):2753–63. Epub 2008/08/08. . 18684966 10.4049/jimmunol.181.4.2753

[100] Leslie M. Cell biology. Beyond clotting: the powers of platelets. Science. 2010;328(5978):562–4. Epub 2010/05/01. 10.1126/science.328.5978.562 . 20430990

[101] Melican K, Boekel J, Mansson LE, Sandoval RM, Tanner GA, Kallskog O, et al Bacterial infection-mediated mucosal signalling induces local renal ischaemia as a defence against sepsis. Cell Microbiol. 2008;10(10):1987–98. Epub 2008/06/14. 10.1111/j.1462-5822.2008.01182.x . 18549455

[102] Hayon Y, Shai E, Varon D, Leker RR. The role of platelets and their microparticles in rehabilitation of ischemic brain tissue. CNS Neurol Disord Drug Targets. 2012;11(7):921–5. Epub 2012/11/08. . 23131157 10.2174/1871527311201070921

[103] Pintucci G, Froum S, Pinnell J, Mignatti P, Rafii S, Green D. Trophic effects of platelets on cultured endothelial cells are mediated by platelet-associated fibroblast growth factor-2 (FGF-2) and vascular endothelial growth factor (VEGF). Thromb Haemost. 2002;88(5):834–42. Epub 2002/11/13. . 12428103

[104] Hasan UA, Trinchieri G, Vlach J. Toll-like receptor signaling stimulates cell cycle entry and progression in fibroblasts. J Biol Chem. 2005;280(21):20620–7. Epub 2005/03/25. 10.1074/jbc.M500877200 . 15788393

[105] Rabbani ZN, Batinic-Haberle I, Anscher MS, Huang J, Day BJ, Alexander E, et al Long-term administration of a small molecular weight catalytic metalloporphyrin antioxidant, AEOL 10150, protects lungs from radiation-induced injury. Int J Radiat Oncol Biol Phys. 2007;67(2):573–80. Epub 2007/01/24. 10.1016/j.ijrobp.2006.09.053 . 17236973 PMC1819401

[106] Lee JH, Park JW. A manganese porphyrin complex is a novel radiation protector. Free Radic Biol Med. 2004;37(2):272–83. Epub 2004/06/19. 10.1016/j.freeradbiomed.2004.04.029 . 15203198

[107] Patchen ML, MacVittie TJ, Williams JL, Schwartz GN, Souza LM. Administration of interleukin-6 stimulates multilineage hematopoiesis and accelerates recovery from radiation-induced hematopoietic depression. Blood. 1991;77(3):472–80. Epub 1991/02/01. . 1991164

[108] Patchen ML, Fischer R, MacVittie TJ. Effects of combined administration of interleukin-6 and granulocyte colony-stimulating factor on recovery from radiation-induced hemopoietic aplasia. Exp Hematol. 1993;21(2):338–44. Epub 1993/02/01. . 7678816

[109] D'Hondt V, Humblet Y, Guillaume T, Baatout S, Chatelain C, Berliere M, et al Thrombopoietic effects and toxicity of interleukin-6 in patients with ovarian cancer before and after chemotherapy: a multicentric placebo-controlled, randomized phase Ib study. Blood. 1995;85(9):2347–53. Epub 1995/05/01. . 7537110

[110] Trinchieri G. Interleukin-12: a cytokine at the interface of inflammation and immunity. Adv Immunol. 1998;70:83–243. Epub 1998/10/02. . 9755338 10.1016/s0065-2776(08)60387-9

