# Supplementary material for: The Toll-Like Receptor 5 Agonist Entolimod Mitigates Lethal Acute Radiation Syndrome in Non-Human Primates
Source: PLoS One. 2015 Sep 14;10(9):e0135388. doi: 10.1371/journal.pone.0135388 (PMC4569586; doi:10.1371/journal.pone.0135388)
Supplement: S1 Fig — (PDF) [file pone.0135388.s001.pdf]

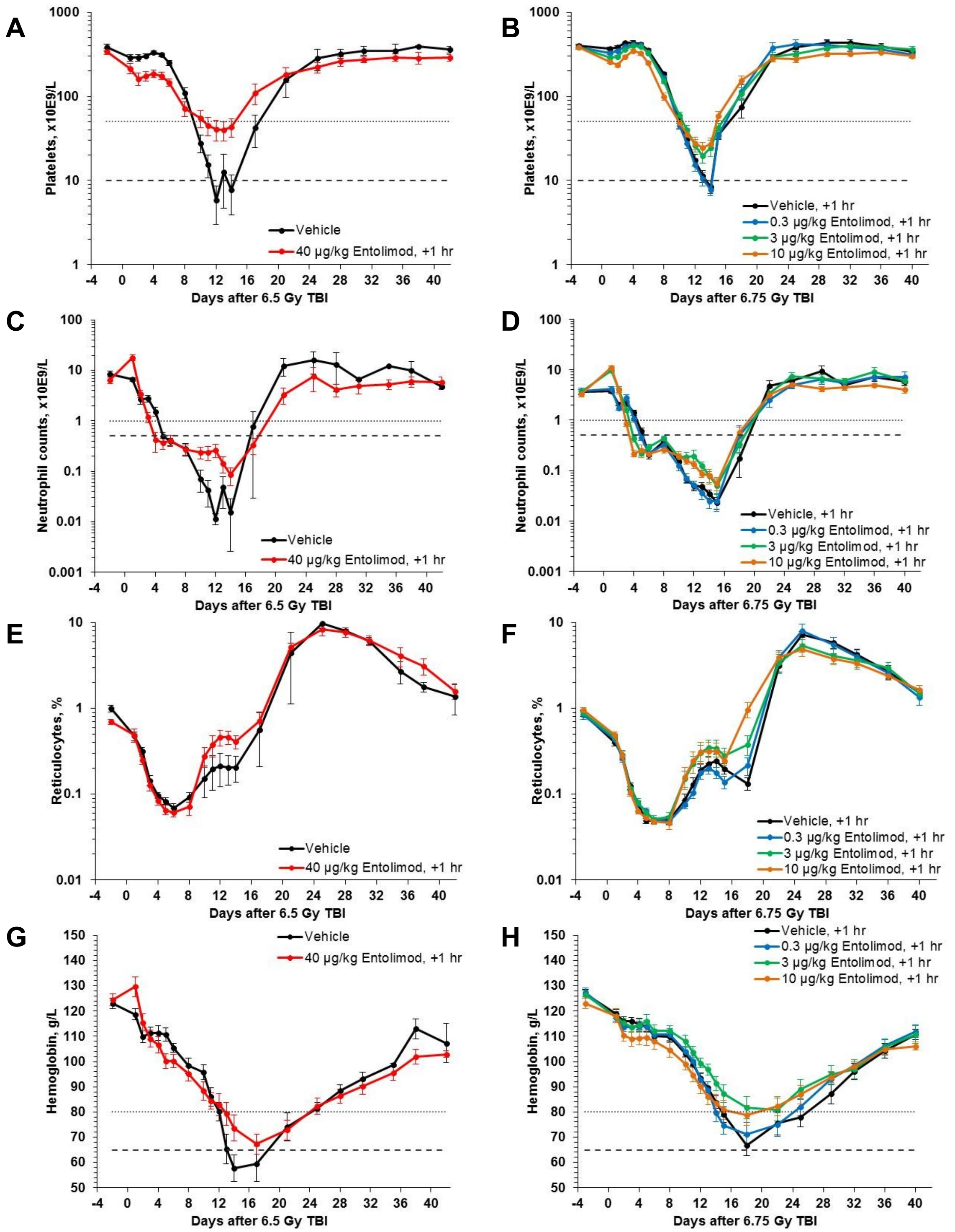

S1 Fig. Accelerated recovery of the peripheral blood cellularity and hemoglobin content in NHPs irradiated with $LD_{50/40}$ or $LD_{75/40}$ of TBI and treated with different doses of entolimod 1 hour later.

A, C, E, G: study Rs-03; N=10. **B, D, F, H:** study Rs-09; N=18. Cytopenia/anemia thresholds: dotted lines - Grade 3 (platelets <50,000/µL; neutrophils <1,000/µL; hemoglobin <80 g/L); dashed lines - Grade 4 (platelets <10,000/µL; neutrophils <500/µL; hemoglobin <65 g/L). Error bars represent standard errors**.**
