# Supplementary material for: The Toll-Like Receptor 5 Agonist Entolimod Mitigates Lethal Acute Radiation Syndrome in Non-Human Primates
Source: PLoS One. 2015 Sep 14;10(9):e0135388. doi: 10.1371/journal.pone.0135388 (PMC4569586; doi:10.1371/journal.pone.0135388)
Supplement: S2 Fig — (PDF) [file pone.0135388.s002.pdf]

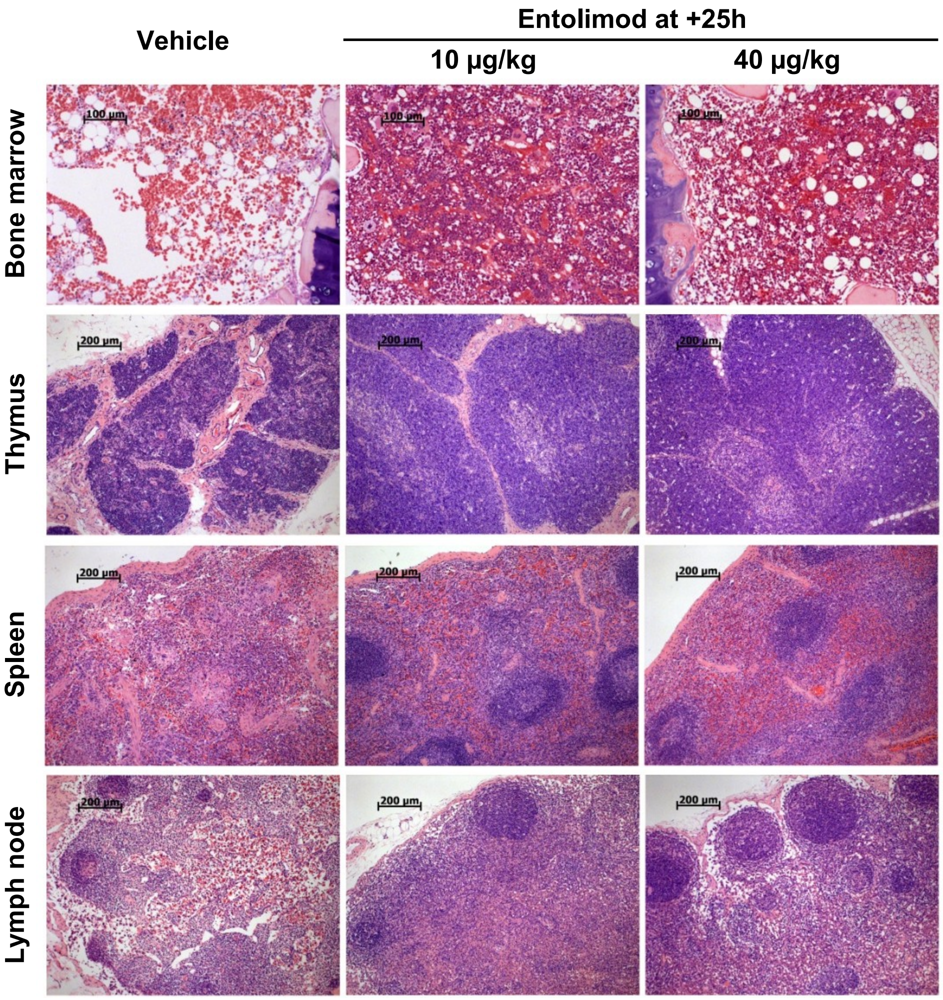


**S2 Fig. Comparable restorative effects of single 10 or 40 μg/kg entolimod treatments given 25 hours after TBI on morphological recovery of hematopoietic and lymphoid organs in NHPs 40 days after irradiation with $LD_{50/40}$ of TBI.**

Representative histological images (hematoxylin-eosin staining) of sternum bone marrow sections, thymuses, spleens and mesenteric lymph nodes from animals that survived to study termination on Day 40 post-TBI (study Rs-14). Scale bars: 100 μm for bone marrow, 200 μm for thymus, spleen, and lymph node.
