# Supplementary material for: The Toll-Like Receptor 5 Agonist Entolimod Mitigates Lethal Acute Radiation Syndrome in Non-Human Primates
Source: PLoS One. 2015 Sep 14;10(9):e0135388. doi: 10.1371/journal.pone.0135388 (PMC4569586; doi:10.1371/journal.pone.0135388)
Supplement: S3 Fig — (PDF) [file pone.0135388.s003.pdf]

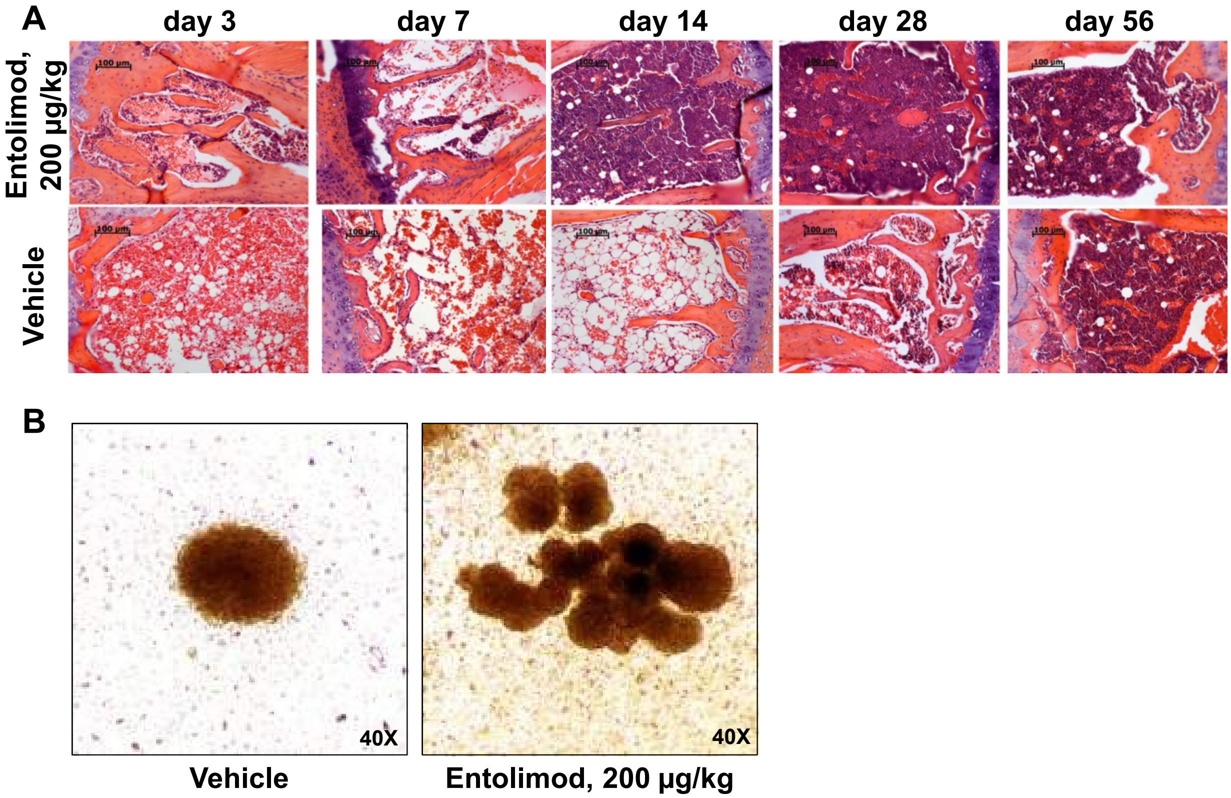


**S3 Fig. Accelerated kinetics of bone marrow regeneration and proliferating phenotype of CFU-GM colonies in entolimod-treated CD2F1 mice after $LD_{50/30}$ of TBI and entolimod treatment.**

Male CD2F1 mice were irradiated with 9 Gy TBI and injected i.m. with vehicle or 200 µg/kg entolimod 25 h later. Groups of 5 mice were sacrificed at the indicated time points for evaluation of histopathology and bone marrow clonogenic potential. **A**: Representative microphotographs of hematoxylin-eosin-stained bone marrow sections. Scale bar – 100 µm. **B**: Appearance of CFU-GM colonies grown from bone marrow collected on day 7 after TBI.
