# Supplementary material for: The Toll-Like Receptor 5 Agonist Entolimod Mitigates Lethal Acute Radiation Syndrome in Non-Human Primates
Source: PLoS One. 2015 Sep 14;10(9):e0135388. doi: 10.1371/journal.pone.0135388 (PMC4569586; doi:10.1371/journal.pone.0135388)
Supplement: S4 Fig — (PDF) [file pone.0135388.s004.pdf]

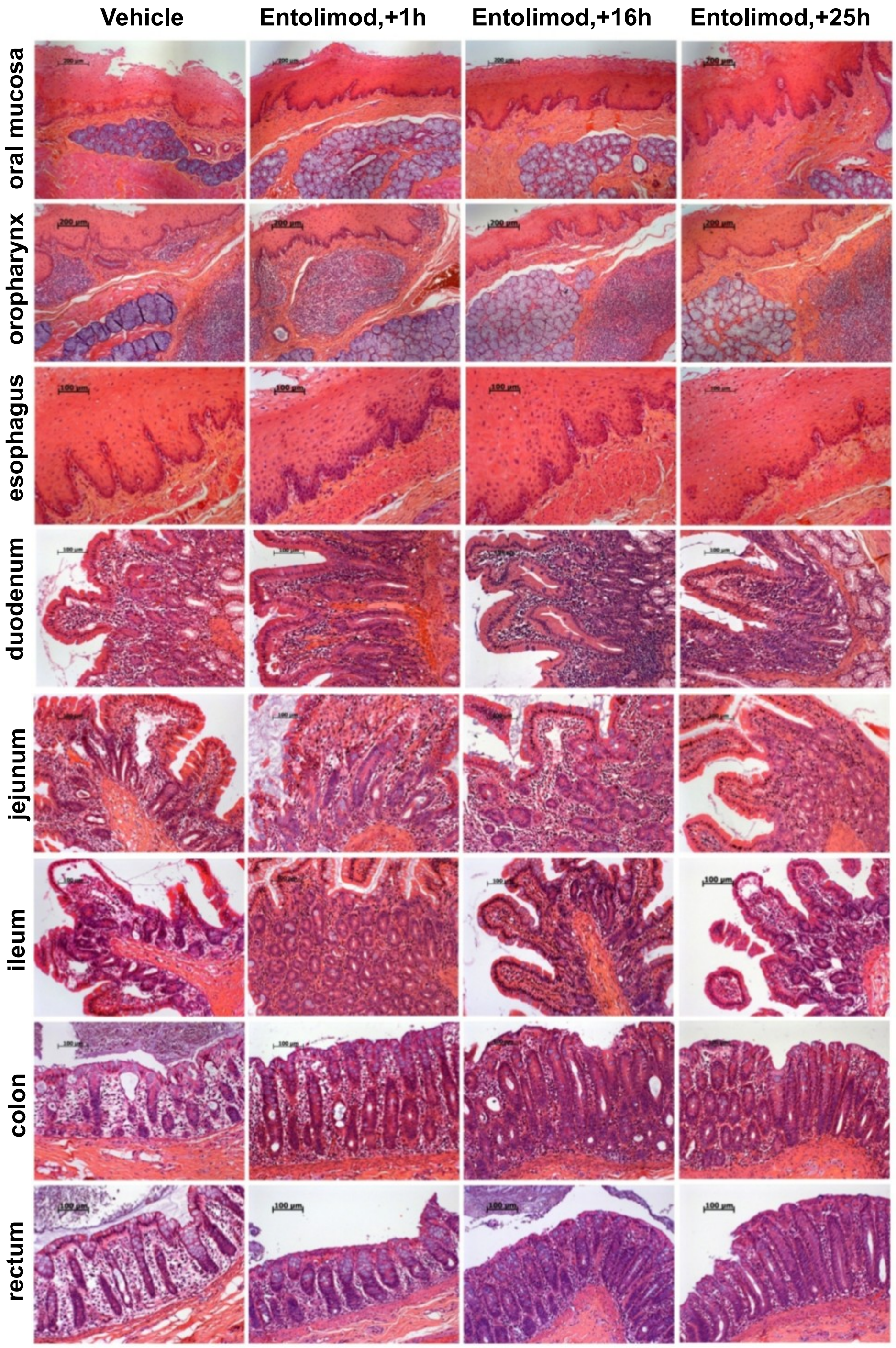

S4 Fig. Improved GI tract morphology in NHPs irradiated with 6.5 Gy TBI and treated with a single injection of entolimod at 1, 16, or 25 hours later.

Rhesus macaques were injected i.m. with vehicle or 40 µg/kg entolimod 1, 16 or 25 h after 6.5 Gy TBI (study Rs-08). Samples were collected on day 5 after TBI for H&E staining. Scale bars - 200 µm for oral mucosa and oropharynx; 100 µm – for all other GI tract segments.
