# Supplementary material for: The Toll-Like Receptor 5 Agonist Entolimod Mitigates Lethal Acute Radiation Syndrome in Non-Human Primates
Source: PLoS One. 2015 Sep 14;10(9):e0135388. doi: 10.1371/journal.pone.0135388 (PMC4569586; doi:10.1371/journal.pone.0135388)
Supplement: S5 Fig — (PDF) [file pone.0135388.s005.pdf]

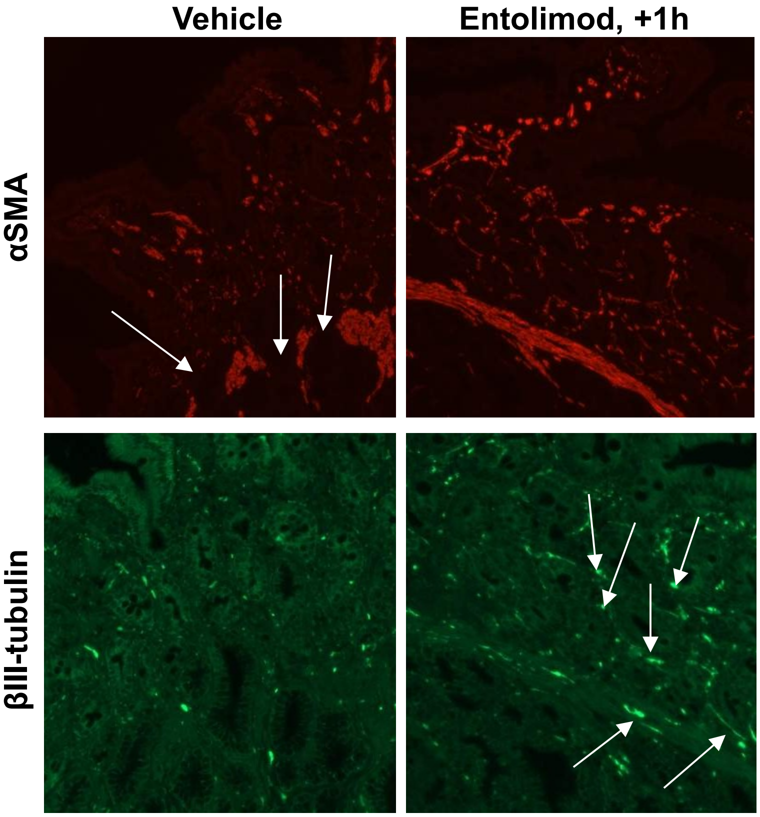

S5 Fig. Improved preservation of intestinal innervation and muscularis mucosae integrity in the GI tract of irradiated NHPs treated with entolimod 1 hour after 6.5 Gy TBI.

Rhesus macaques were injected i.m. with vehicle or 40 μg/kg entolimod 1 h after 6.5 Gy TBI and duodenum samples were collected 5 days later (study Rs-08). Upper panels: arrows point to disruptions of muscularis mucosae (red) - present mostly in vehicle-treated NHPs. Bottom panels: arrows point to axons and neural termini (green) in the cryptal area of the small intestine - more abundant in entolimod-treated NHPs.
