# Supplementary material for: The Toll-Like Receptor 5 Agonist Entolimod Mitigates Lethal Acute Radiation Syndrome in Non-Human Primates
Source: PLoS One. 2015 Sep 14;10(9):e0135388. doi: 10.1371/journal.pone.0135388 (PMC4569586; doi:10.1371/journal.pone.0135388)
Supplement: S6 Fig — (PDF) [file pone.0135388.s006.pdf]

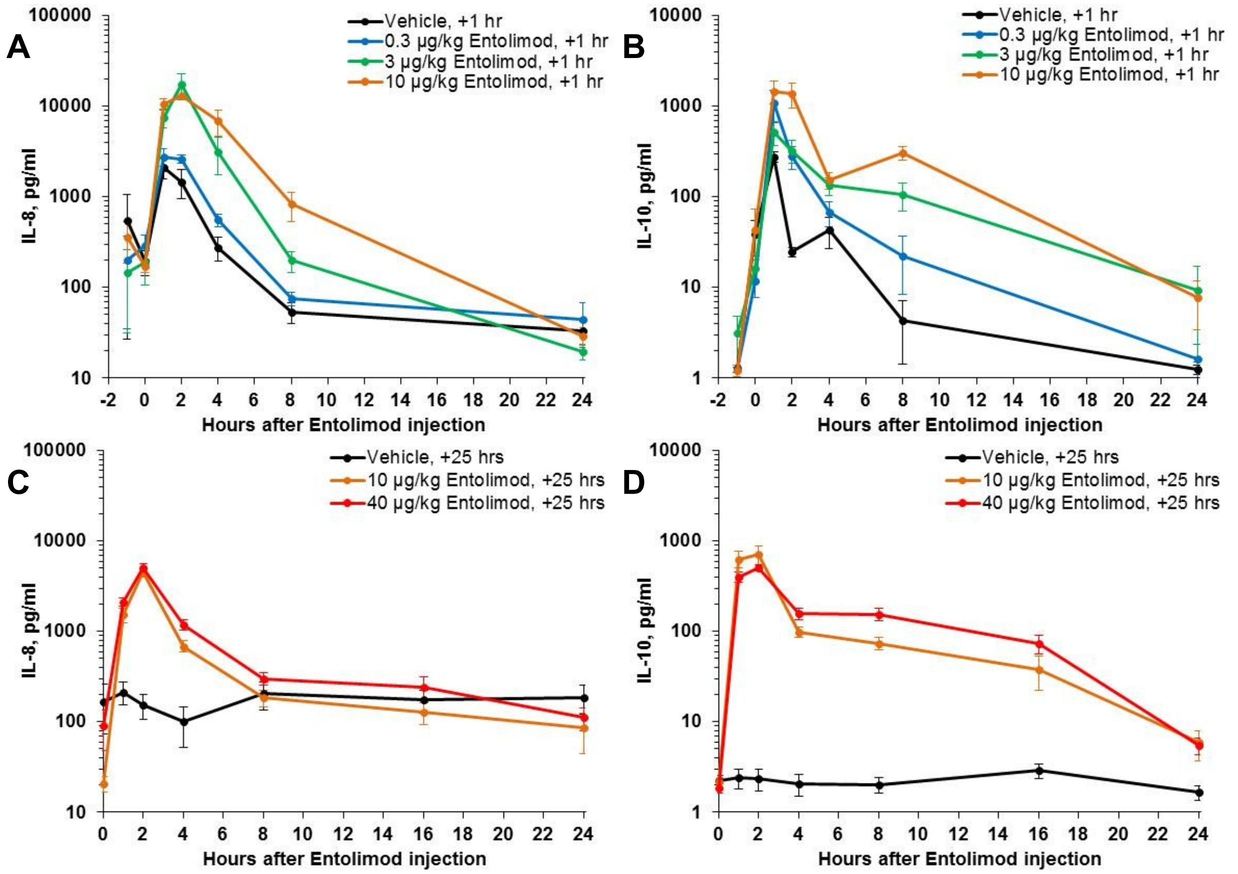

S6 Fig. Effect of single dose entolimod treatment on IL-8 and IL-10 levels in the peripheral blood of NHPs irradiated with $LD_{50/40}$ or $LD_{75/40}$ doses of TBI.

A, B: Effects of different entolimod doses administered 1 h after TBI (study Rs-09; N=18). **C, D:** Effect of different entolimod doses administered 25 h after TBI (study Rs-14; N=10). Error bars represent standard errors.
