# Supplementary material for: The Toll-Like Receptor 5 Agonist Entolimod Mitigates Lethal Acute Radiation Syndrome in Non-Human Primates
Source: PLoS One. 2015 Sep 14;10(9):e0135388. doi: 10.1371/journal.pone.0135388 (PMC4569586; doi:10.1371/journal.pone.0135388)
Supplement: S7 Fig — (PDF) [file pone.0135388.s007.pdf]

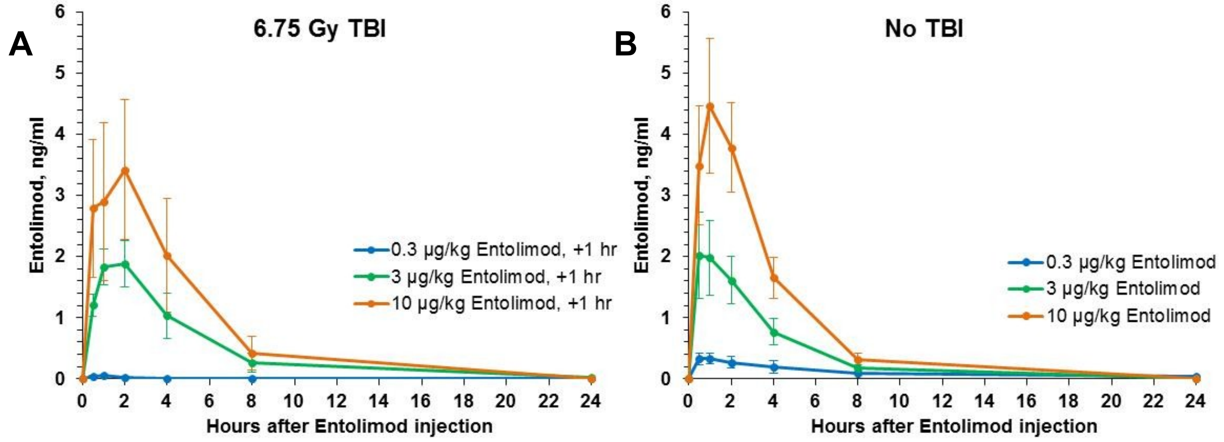

S7 Fig. Entolimod concentrations in the peripheral blood of NHPs (irradiated with $LD_{50/40}$ (6.75 Gy) TBI or non-irradiated) at different times after single intramuscular injection of the indicated drug doses.

A: Entolimod levels after injection of different doses 1 h after TBI (study Rs-09; N=18). **B:** Entolimod levels after injection of the same dose levels in non-irradiated NHPs (study 04-Rs-04; N=6). This study is not described in the current manuscript and data are shown for comparison. Error bars represent standard errors.
