# Supplementary material for: The Toll-Like Receptor 5 Agonist Entolimod Mitigates Lethal Acute Radiation Syndrome in Non-Human Primates
Source: PLoS One. 2015 Sep 14;10(9):e0135388. doi: 10.1371/journal.pone.0135388 (PMC4569586; doi:10.1371/journal.pone.0135388)
Supplement: S1 Methods — (PDF) [file pone.0135388.s008.pdf]

Supporting Materials and Methods

Semi-quantitative scoring of hematopoietic (HP) and gastrointestinal (GI) organ morphology on histological sections

The extent of morphologic alterations in hematological, immune and GI organs was assessed by blind semi-quantitative evaluation of the histological sections (2-3 slides/location/animal) using scoring systems developed for each specific morphological location and based on evaluation of multiple morphological parameters.

Bone marrow evaluation took into account the number of HP cells in the sections and the nature of HP elements present (granulocytic, erythroid, and megakaryocytic lineages). The appearance of HP cells themselves was evaluated (e.g., whether they were spread and in contact with each other forming regular groups, plates, or islands or whether they were rounded and detached from each other as a sign of injury). Scores were as follows: 0: total aplasia; 0 to <1: pronounced atrophy with absence or minimal presence of HP cells; 1 to <2: pronounced atrophy but with HP cells present albeit in numbers much lower than normal; 2 to <3: moderate to slight atrophy; 3 to <4: slight atrophy; 4: normal morphology.

Spleen evaluation took into account presence of HP elements (lymphocytes, erythrocytes, granulocytes and macrophages) in the red and white pulp as well as presence of typical structural elements such as lymphoid follicles and periarteriolar lymphoid sheath (PALS) zones. Scores were as follows: 0: total aplasia; 1: pronounced atrophy with minimal emerging white pulp lymphoid repopulation and better appearance of the red pulp; 2: moderate changes in spleen morphology with the white and red pulp approximately half-way recovered; 3: structure almost normal with slight hypocellularity of the red and white pulp; 4: normal morphology.

Thymus evaluation took into account presence of lymphocytes and typical structural elements such as the cortical and medulla zones. Scores were as follows: 0: total lymphoid depopulation; 1: pronounced atrophy with minimal presence of residual lymphocytes in the medullar zone and morphologically non-distinct cortex. 2: moderate changes with lymphocytes visible both in the cortex and the medulla, but with a poorly defined boundary between these

zones. 3: near-normal structure but with decreased cortex to medulla ratio; lymphoid hypocellularity, macrophages present in the cortex. 4: normal morphology.

Lymph node evaluation took into account presence of lymphocytes and typical structural features such as the medulla, cortex, and paracortex. Scores were as follows: 0: total lymphoid depopulation; 1: pronounced atrophy with minimal presence of lymphocytes in any structural zone; 2: atrophy but with clear follicular structures and lymphocytes present in the paracortex and medulla. 3: minimal atrophy with slight lymphoid hypocellularity; 4: normal.

Gastrointestinal tract evaluation took into account radiation-induced changes in: (a) *villi and surface epithelium* – height and width of villi of the small intestines, appearance and number of surface enterocytes, goblet cells and their nuclei (in small and large intestines); (b) *crypts* (and Brunner's glands in the duodenum) - depth, size and shape of crypts (and Brunner's glands in the duodenum); changes in epithelial cells; presence of apoptotic bodies; presence of mitotic figures and other signs of regeneration and differentiation; luminal migration of nuclei in epithelial cells; loss of goblet cells; presence of cryptitis and crypt abscesses; atrophy and loss of glands; (c) *lamina propria* (stroma, transitory and lymphoid elements and submucosa) - state of the lamina propria and transitory cells in it, state of lymphoid accumulations associated with the mucosa, presence of edema, blood vessel congestion and hemorrhage. Scores were as follows: 0: severely abnormal; 1: markedly abnormal; 2: moderately abnormal; 3: mildly abnormal; 4: normal morphology.
