# Supplementary material for: The Toll-Like Receptor 5 Agonist Entolimod Mitigates Lethal Acute Radiation Syndrome in Non-Human Primates
Source: PLoS One. 2015 Sep 14;10(9):e0135388. doi: 10.1371/journal.pone.0135388 (PMC4569586; doi:10.1371/journal.pone.0135388)
Supplement: S1 Table — (PDF) [file pone.0135388.s009.pdf]

S1 Table. Incidence and duration of Grade 4 neutropenia (neutrophil count <500 cells/µL) in lethally irradiated NHPs treated with vehicle or entolimod

Study	Irradiation dose	Entolimod dose, µg/kg	Injection time(s) relative to TBI, h	Group size (n)	Mean % live days ± SE with Grade 4 neutropenia	P-value[A]	Incidence of Grade 4 neutropenia	P-value[B]
Rs-03	~LD$_{75/40}$ (6.5 Gy)[C]	0 (vehicle)	+1	10	56%±6%	-	100%	-
		40	+1	10	33%±6%	**0.01**	90%	>0.05
Rs-06	~LD$_{75/40}$ (6.5 Gy)[C]	0 (vehicle)	+16	8	60%±6%	-	100%	-
		40	+16	12	47%±6%	0.13	100%	>0.05
		40	+25	10	44%±6%	0.08	100%	>0.05
		40	+48	12	51%±8%	0.35	100%	>0.05
Rs-09	~LD$_{50/40}$ (6.75 Gy)[D]	0 (vehicle)	+1	18	51%±4%	-	100%	-
		0.3	+1	18	48%±5%	0.65	100%	>0.05
		3	+1	18	44%±4%	0.22	100%	>0.05
		10	+1	18	38%±2%	**0.01**	100%	>0.05
Rs-14	~LD$_{50/40}$ (6.75 Gy)[D]	0 (vehicle)	+25	10	55%±6%	-	100%	-
		10	+25	10	37%±2%	**0.01**	100%	>0.05
		40	+25	10	42%±5%	0.11	100%	>0.05
Pooled vehicle vs. ≥10 µg/kg entolimod, +25 h	~LD$_{50\text{-}75/40}$ (6.5-6.75 Gy)	0 (vehicle) [E]	+1 - +25	46	55%±3%	-	100%	-
		≥10[F]	+25	30	41%±3%	**0.001**	100%	>0.05

[A] P-value by Student's t-test (two-tailed) against vehicle groups in individual studies or in pooled group analysis

[B] P-value by Fisher's exact test (two-tailed) against vehicle groups in individual studies or in pooled group analysis

[C] Source I: Sichuan Atomic Energy Institute, cylindrical bundle of Co-60 rods

[D] Source II: Sichuan Atomic Energy Institute, vertical array of Co-60 rods

[E] Vehicle-treated animals from studies Rs-03, Rs-06, Rs-09, and Rs-14

[F] Entolimod-treated animals from studies Rs-06 and Rs-14
