# Supplementary material for: The Toll-Like Receptor 5 Agonist Entolimod Mitigates Lethal Acute Radiation Syndrome in Non-Human Primates
Source: PLoS One. 2015 Sep 14;10(9):e0135388. doi: 10.1371/journal.pone.0135388 (PMC4569586; doi:10.1371/journal.pone.0135388)
Supplement: S2 Table — (PDF) [file pone.0135388.s010.pdf]

S2 Table. Incidence and duration of absolute neutropenia (neutrophil count <10 cells/µL) in lethally irradiated NHPs treated with vehicle or entolimod

Study	Irradiation dose	Entolimod dose, µg/kg	Injection time(s) relative to TBI, h	Group size (n)	Mean % live days ± SE with absolute neutropenia	P-value[A]	Incidence of absolute neutropenia	P-value[B]
Rs-03	~$LD_{75/40}$ (6.5 Gy)[C]	0 (vehicle)	+1	10	16%±4%	-	80%	-
		40	+1	10	1%±1%	**0.002**	0%	**0.001**
Rs-06	~$LD_{75/40}$ (6.5 Gy)[C]	0 (vehicle)	+16	8	15%±3%	-	88%	-
		40	+16	12	3%±2%	**0.01**	25%	**0.02**
		40	+25	10	6%±2%	**0.03**	50%	>0.05
		40	+48	12	8%±3%	0.11	50%	>0.05
Rs-09	~$LD_{50/40}$ (6.75 Gy)[D]	0 (vehicle)	+1	18	10%±2%	-	78%	-
		0.3	+1	18	11%±3%	0.74	72%	>0.05
		3	+1	18	7%±2%	0.39	50%	>0.05
		10	+1	18	4%±1%	**0.02**	56%	>0.05
Rs-14	~$LD_{50/40}$ (6.75 Gy) [D]	0 (vehicle)	+25	10	6%±2%	-	50%	-
		10	+25	10	2%±1%	0.19	30%	>0.05
		40	+25	10	5%±3%	0.84	50%	>0.05
Pooled vehicle vs. ≥10 µg/kg entolimod, +25 h	~$LD_{50\text{-}75/40}$ (6.5-6.75 Gy)	0 (vehicle) [E]	+1 - +25	46	11%±1%	-	74%	-
		≥10[F]	+25	30	4%±1%	**0.0003**	43%	**0.01**

[A] P-value by Student's t-test (two-tailed) against vehicle groups in individual studies or in pooled group analysis

[B] P-value by Fisher's exact test (two-tailed) against vehicle groups in individual studies or in pooled group analysis

[C] Source I: Sichuan Atomic Energy Institute, cylindrical bundle of Co-60 rods

[D] Source II: Sichuan Atomic Energy Institute, vertical array of Co-60 rods

[E] Vehicle-treated animals from studies Rs-03, Rs-06, Rs-09, and Rs-14

[F] Entolimod-treated animals from studies Rs-06 and Rs-14
