# Supplementary material for: The Toll-Like Receptor 5 Agonist Entolimod Mitigates Lethal Acute Radiation Syndrome in Non-Human Primates
Source: PLoS One. 2015 Sep 14;10(9):e0135388. doi: 10.1371/journal.pone.0135388 (PMC4569586; doi:10.1371/journal.pone.0135388)
Supplement: S4 Table — (PDF) [file pone.0135388.s012.pdf]

S4 Table. Incidence and duration of Grade 4 anemia (hemoglobin level <65g/L) in lethally irradiated NHPs treated with vehicle or entolimod

Study	Irradiation dose	Entolimod dose, µg/kg	Injection time(s) relative to TBI, h	Group size (n)	Mean % live days ± SE with Grade 4 anemia	P-value[A]	Incidence of Grade 4 anemia	P-value[B]
Rs-03	~$LD_{75/40}$ (6.5 Gy [C]	0 (vehicle)	+1	10	11%±3%	-	70%	-
		40	+1	10	6%±3%	0.29	20%	>0.05
Rs-06	~$LD_{75/40}$ (6.5 Gy)[C]	0 (vehicle)	+16	8	8%±5%	-	25%	-
		40	+16	12	2%±2%	0.25	8%	>0.05
		40	+25	10	6%±4%	0.78	20%	>0.05
		40	+48	12	1%±1%	0.20	0%	>0.05
Rs-09	~$LD_{50/40}$ (6.75 Gy)[D]	0 (vehicle)	+1	18	9%±3%	-	44%	-
		0.3	+1	18	7%±2%	0.49	33%	>0.05
		3	+1	18	6%±3%	0.50	17%	>0.05
		10	+1	18	6%±3%	0.41	17%	>0.05
Rs-14	~$LD_{50/40}$ (6.75 Gy)[D]	0 (vehicle)	+25	10	12%±4%	-	60%	-
		10	+25	10	1%±1%	**0.01**	20%	>0.05
		40	+25	10	1%±1%	**0.02**	10%	>0.05
Pooled vehicle vs. ≥10 µg/kg entolimod, +25 h	~$LD_{50-75/40}$ (6.5-6.75 Gy)	0 (vehicle) [E]	+1 - +25	46	10%±2%	-	50%	-
		≥10[F]	+25	30	3%±2%	**0.003**	17%	**0.004**

[A] P-value by Student's t-test (two-tailed) against vehicle group

[B] P-value by Fisher's exact test (two-tailed) against vehicle group

[C] Source I: Sichuan Atomic Energy Institute, cylindrical central set of Co60 rods

[D] Source II: Sichuan Atomic Energy Institute, vertical array of Co60 rods

[E] Vehicle-treated animals from studies Rs-03, Rs-06, Rs-09, and Rs-14

[F] Entolimod-treated animals from studies Rs-06 and Rs-14
