# Supplementary material for: The Toll-Like Receptor 5 Agonist Entolimod Mitigates Lethal Acute Radiation Syndrome in Non-Human Primates
Source: PLoS One. 2015 Sep 14;10(9):e0135388. doi: 10.1371/journal.pone.0135388 (PMC4569586; doi:10.1371/journal.pone.0135388)
Supplement: S5 Table — (PDF) [file pone.0135388.s013.pdf]

S5 Table. Semi-quantitative histological evaluation of hematopoietic/lymphoid organs from NHPs that survived to day 40 after 6.75 Gy TBI followed by vehicle or entolimod treatment (study Rs-14)

Organ/tissue	Mean score [A] ± SE			P-value vs. vehicle [B]	
	Vehicle (N=4)	Entolimod, +25h		Entolimod, +25h	
		10 µg/kg (N=10)	40 µg/kg (N=8)	10 µg/kg	40 µg/kg
**Bone marrow**	2.2±0.7	3.7±0.1	3.9±0.0	0.1	0.1
**Thymus** [C]	2.1±0.1	3.5±0.2	3.8±0.1	**0.007**	**0.002**
**Spleen**	1.0±0.3	2.7±0.2	3.6±0.1	**0.004**	**0.001**
**Lymph node**	1.2±0.2	2.3±0.3	3.7±0.1	**0.007**	**0.0003**

[A] Scoring was performed based on a 5-grade scale developed for each organ: 0 – total aplasia; 1 – pronounced atrophy, 2 – moderate atrophy, 3 – slight atrophy, close to normal morphology; 4 – normal morphology. Scoring criteria for individual organs are described in Supplementary Methods.

[B] Student's t-test vs. vehicle, 2-tailed.

[C] Due to thymus atrophy, fewer thymus samples were evaluated compared to other organs (N = 2, 4, and 6 for vehicle, 10 µg/kg, and 40 µg/kg groups, respectively).
