# Supplementary material for: The Toll-Like Receptor 5 Agonist Entolimod Mitigates Lethal Acute Radiation Syndrome in Non-Human Primates
Source: PLoS One. 2015 Sep 14;10(9):e0135388. doi: 10.1371/journal.pone.0135388 (PMC4569586; doi:10.1371/journal.pone.0135388)
Supplement: S6 Table — (PDF) [file pone.0135388.s014.pdf]

S6 Table. Semi-quantitative histological evaluation of GI tract segments from NHPs that survived to day 40 after 6.5 Gy TBI and vehicle or entolimod treatment (study Rs-06)

Organ/tissue	Mean score [A] ± SE				P-value vs. vehicle [B]		
	Vehicle (N=2)	Entolimod, 40 µg/kg			Entolimod, 40 µg/kg		
		+16 h (N=8)	+25 h (N=7)	+48 h (N=8)	16 h	25 h	48 h
**Duodenum**	2.4±0.1	3.1±0.2	2.9±0.2	3.2±0.1	**0.03**	0.07	**0.01**
**Jejunum**	2.8±0.3	3.8±0.1	3.6±0.1	3.6±0.1	0.21	0.24	0.25
**Ileum**	3.3±0.3	3.6±0.1	3.6±0.1	3.7±0.1	0.35	0.36	0.31
**Cecum**	2.8±0.2	3.5±0.1	3.6±0.1	3.4±0.1	0.11	**0.05**	0.10
**Colon**	3.6±0.3	3.5±0.1	3.3±0.1	3.5±0.1	0.71	0.46	0.76
**Rectum**	3.0±0.7	3.0±0.2	3.0±0.2	3.4±0.2	0.98	0.98	0.64

[A] 0: severely abnormal; 1: markedly abnormal; 2: moderately abnormal; 3: mildly abnormal; 4: normal (see Supplementary Methods). Sample-average scores were calculated for each sample over evaluated histological sub-structures (villi/epithelium, crypts, lamina propria/submucosa, Brunner's glands). Mean sample-average scores per group are shown.

[B] Student's t-test vs. vehicle, 2-tailed.
