# Supplementary material for: The Toll-Like Receptor 5 Agonist Entolimod Mitigates Lethal Acute Radiation Syndrome in Non-Human Primates
Source: PLoS One. 2015 Sep 14;10(9):e0135388. doi: 10.1371/journal.pone.0135388 (PMC4569586; doi:10.1371/journal.pone.0135388)
Supplement: S7 Table — (PDF) [file pone.0135388.s015.pdf]

S7 Table. Layout of studies dedicated to assessment of entolimod effects on GI tract histopathology in the course of ARS

Study Number	Irradiation dose and source	Entolimod dose, µg/kg	Injection time relative to TBI, h	Group size	Timing of scheduled euthanasia (after TBI)
Rs-04	~$LD_{75/40}$ (6.5 Gy); Co60[A]	0 (vehicle)	+1	2	8h
		40	+1	2	8h
		0 (vehicle)	+1	2	5d
		40	+1	2	5d
Rs-08	~$LD_{75/40}$ (6.5 Gy); Co60[A]	0 (vehicle)	+1	2	8h
		3	+1	2	8h
		10	+1	2	8h
		40	+1	4	8h
		100	+1	2	8h
		200	+1	2	8h
		0 (vehicle)	+1	2	5d
		3	+1	2	5d
		10	+1	2	5d
		40	+1	4	5d
		100	+1	2	5d
		200	+1	2	5d
		40	+16	2	5d
		40	+25	2	5d
Rs-22	>$LD_{95/40}$ (11 Gy); LINAC[B]	0 (vehicle)	+4	4	7d[C]
		40	+4	4	7d[C]

[A] Source I: Sichuan Atomic Energy Institute, cylindrical bundle of cobalt rods

[B] Source III: UIC TRL, 6 MV LINAC source (Varian Clinac 2100EX)

[C] 10 mg/kg EdU, i.v. 1 h before euthanasia
