# Supplementary material for: The Toll-Like Receptor 5 Agonist Entolimod Mitigates Lethal Acute Radiation Syndrome in Non-Human Primates
Source: PLoS One. 2015 Sep 14;10(9):e0135388. doi: 10.1371/journal.pone.0135388 (PMC4569586; doi:10.1371/journal.pone.0135388)
Supplement: S8 Table — (PDF) [file pone.0135388.s016.pdf]

S8 Table. Semi-quantitative histological evaluation of GI tract sub-structures on day 7 after 11 Gy TBI and vehicle or 40 μg/kg entolimod treatment at 4 h post-TBI (study Rs-22, N=4)

Intestinal structure	Vehicle score [A], mean±SE	40 μg/kg Entolimod score [A], mean±SE	T-test P-value (E vs. V) [B]
**MALT [C]**	1.0±0.03	1.4±0.04	**<0.0001**
**Lamina Propria**	1.0±0.04	1.3±0.03	**<0.0001**
**Surface Epithelium**	1.1±0.06	1.4±0.03	**<0.0001**
**Crypts**	1.1±0.07	1.4±0.06	**<0.0001**
**Villi**	0.9±0.09	1.3±0.06	**<0.0001**

[A] 0: severely abnormal; 1: markedly abnormal; 2: moderately abnormal; 3: mildly abnormal; 4: normal (see Supplementary Methods).

[B] Student's t-test of 40 μg/kg entolimod (E) vs. vehicle (V) scores, 2-tailed.

[C] Mucosa-associated lymphoid tissue.
